# Tumor Progression Locus 2 Promotes Induction of IFNλ, Interferon Stimulated Genes and Antigen-Specific CD8^+^ T Cell Responses and Protects against Influenza Virus

**DOI:** 10.1371/journal.ppat.1005038

**Published:** 2015-08-04

**Authors:** Teneema Kuriakose, Ralph A. Tripp, Wendy T. Watford

**Affiliations:** Department of Infectious Diseases, College of Veterinary Medicine, University of Georgia, Athens, Georgia, United States of America; St. Jude Children's Research Hospital, UNITED STATES

## Abstract

Mitogen-activated protein kinase (MAP) cascades are important in antiviral immunity through their regulation of interferon (IFN) production as well as virus replication. Although the serine-threonine MAP kinase tumor progression locus 2 (Tpl2/MAP3K8) has been implicated as a key regulator of Type I (IFNα/β) and Type II (IFNγ) IFNs, remarkably little is known about how Tpl2 might contribute to host defense against viruses. Herein, we investigated the role of Tpl2 in antiviral immune responses against influenza virus. We demonstrate that Tpl2 is an integral component of multiple virus sensing pathways, differentially regulating the induction of IFNα/β and IFNλ in a cell-type specific manner. Although Tpl2 is important in the regulation of both IFNα/β and IFNλ, only IFNλ required Tpl2 for its induction during influenza virus infection both *in vitro* and *in vivo*. Further studies revealed an unanticipated function for Tpl2 in transducing Type I IFN signals and promoting expression of interferon-stimulated genes (ISGs). Importantly, Tpl2 signaling in nonhematopoietic cells is necessary to limit early virus replication. In addition to early innate alterations, impaired expansion of virus-specific CD8^+^ T cells accompanied delayed viral clearance in *Tpl2^-/-^* mice at late time points. Consistent with its critical role in facilitating both innate and adaptive antiviral responses, Tpl2 is required for restricting morbidity and mortality associated with influenza virus infection. Collectively, these findings establish an essential role for Tpl2 in antiviral host defense mechanisms.

## Author Summary

Influenza viruses infect millions of people annually causing significant morbidity, mortality and socio-economic burdens. Host immune responses against influenza virus are initiated upon virus recognition by specific intracellular receptors. Signals relayed from these receptors trigger various signaling cascades, which induce an antiviral immune response to control infection. Herein, we identified the serine-threonine kinase tumor progression locus 2 (Tpl2) as an essential component of virus sensing pathways, regulating induction of interferons (IFNs) and IFN-induced antiviral genes that restrict virus replication. We also demonstrate that Tpl2 is necessary for generation of effector CD8^+^ T cells, which are required for viral clearance from infected lungs. Consistent with the impaired antiviral responses, Tpl2-deficient mice are defective in controlling virus replication and succumb to influenza virus infection with a normally low pathogenicity strain. Thus, our study identifies Tpl2 as a host factor that integrates antiviral innate and adaptive responses to restrict morbidity and mortality during influenza virus infection.

## Introduction

Mitogen-activated protein kinase (MAP) cascades represent major intracellular signaling pathways activated in response to a variety of external stimuli. Their activation during infection leads to transcriptional induction of immune and inflammatory mediators. Although MAP kinase signaling is important in eliciting host protective responses, many viruses are known to utilize these pathways directly for their replication [1]. Activation of MAP kinases occurs during virus recognition by pattern recognition receptors (PRRs) like toll-like receptors (TLRs) and RIG-I-like RNA helicases (RLH) [2]. Virus sensing by these receptors activates multiple intracellular signaling cascades including NFκB, MAP kinase and IRF pathways that coordinately regulate induction of interferons (IFNs) which are important mediators of antiviral resistance [3]. Among the MAP kinases, tumor progression locus 2 (Tpl2/MAP3K8), a MAP3 kinase, plays an important role in regulating IFN production by promoting the ERK-dependent induction of *c-fos*, a component of AP-1 heterodimeric transcription factors [4]. While Tpl2 is required for IFNα production by plasmacytoid dendritic cells (pDCs) and IFNγ secretion by CD4^+^ T cells, it is a potent negative regulator of IFNβ in macrophages and DCs [4, 5]. Despite being identified as a major regulator of both Type I (IFNα/β) and Type II (IFNγ) IFNs, Tpl2 regulation of Type III IFNs (IFNλs) has not been investigated so far.

Tpl2 was initially identified as an oncogene that induces T cell lymphomas in rodents [6], but more recent studies have established its criticality in regulating both innate and adaptive immune responses via its cell type- and stimulus-specific activation of the MEK-ERK MAPK pathway. Tpl2 regulates signal transduction and cellular responses downstream of TLRs, cytokine receptors, antigen receptors and G protein-coupled receptors [4, 7–9]. In addition to IFNs, Tpl2 also regulates the production of other prominent immune mediators like TNFα, IL-1β IL-10, IL-12 and COX-2 [4, 10–12]. Consequently, Tpl2 is essential for mounting effective immune responses during infections, and *Tpl2* ^*-/-*^ mice are more susceptible to *Toxoplasma gondii* [5], *Listeria monocytogenes* [11], *Mycobacterium tuberculosis* [13] and *Group B Streptococcus* [14]. Surprisingly, there is still limited and contradictory information about how Tpl2 contributes to host defense against viruses. Early studies reported normal cytotoxic T cell responses against lymphocytic choriomeningitis virus [10] and resistance to mouse cytomegalovirus infection [14]. However, another study delineating the signaling circuitry in virus sensing pathways implicated Tpl2 as a key regulator of both inflammatory and antiviral gene induction in response to model viral ligands [15]. A recent study also reported increased replication of vesicular stomatitis virus in Tpl2-deficient mouse embryonic fibroblasts (MEFs) [16].

We recently demonstrated that among the TLRs implicated in virus sensing (TLRs 3, 7 and 9), Tpl2 plays a prominent role in TLR7 signaling [17]. In this study, we investigated Tpl2’s regulation of antiviral responses using a murine model of influenza virus infection, which relies upon TLR7 for virus sensing [18], ERK MAP kinase for virus replication [19] and where both IFNα/β and IFNλ are host protective [20]. Our experiments demonstrate positive regulation of IFNλ and cell-type specific regulation of IFNα/β production in Tpl2-deficient cells following stimulation with model viral ligands that trigger influenza virus sensing receptors, TLR7 or RIG-I. However, during influenza virus infection, IFNλ uniquely required Tpl2 for its induction. Moreover, Tpl2 is involved in IFN signaling, regulating ERK activation and STAT1^ser727^ phosphorylation, and is required for proper induction of antiviral IFN-stimulated genes (ISGs). Impaired ISG induction coupled with reduced antigen-specific CD8^+^ T cells resulted in failure to control virus replication and significant morbidity and mortality of *Tpl2* ^*-/-*^ mice to an otherwise low pathogenicity strain of influenza virus. Collectively, this study establishes Tpl2 as a host factor that integrates antiviral responses to control influenza virus infection.

## Results

### Tpl2 ablation enhances virus replication and inflammatory responses during influenza infection

**Fig 1:**
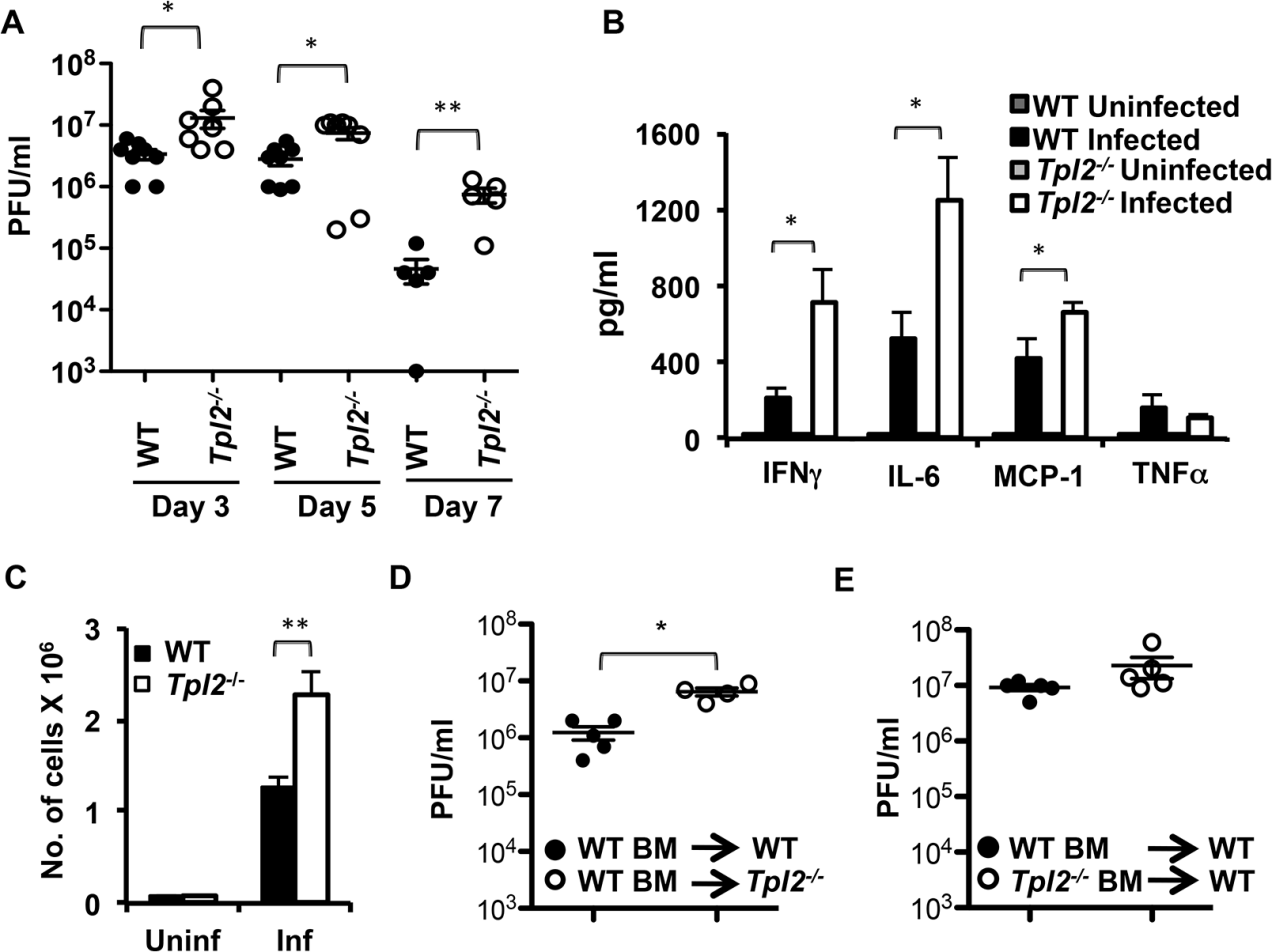
Tpl2 ablation enhances virus replication and inflammatory responses during influenza infection. **(A)** WT and *Tpl2* ^*-/-*^ mice were intranasally infected with 10^4^ pfu of X31 virus, and lung viral titers were enumerated by plaque assays; n = 8 (D3 and D5) or n = 5 (D7). WT and *Tpl2* ^*-/-*^ mice were infected with 10^4^ pfu of X31 virus, and the cytokine levels **(B)** and number of cells recovered **(C)** in BALF were measured on D7 pi; n = 6 uninfected and 10 (WT) and 8 (*Tpl2* ^*-/-*^) infected. **(D-E)** Chimeric mice were intranasally infected with 10^4^ pfu of X31 virus, and lung viral titers were enumerated by plaque assays D3 pi. * indicates *p*<0.05, ** indicates *p*<0.01.

To determine whether Tpl2 regulates influenza virus replication, wild type (WT) and *Tpl2* ^*-/-*^ mice were infected with 10^4^ plaque forming units (pfu) of mouse-adapted influenza virus A/HK-X31(H3N2) (X31), and viral titers in the lungs were evaluated on days 3, 5 and 7 post infection (pi). The average lung viral titers were significantly higher in *Tpl2* ^*-/-*^ mice compared to WT mice at all time points examined (Fig 1A). Notably, average viral titers were more than ten-fold higher in *Tpl2* ^*-/-*^ lungs at day 7 pi. This increase in virus replication was also observed in littermate control mice (S1 Fig). In addition to viral titers, early proinflammatory cytokines, except TNFα were significantly higher in the BALF of *Tpl2* ^*-/-*^ mice compared to WT mice (Fig 1B). Consistent with increased virus replication, total cellular infiltration was also significantly increased in the lungs of *Tpl2* ^*-/-*^ mice at day 7 pi (Fig 1C). The increased lung viral titers in *Tpl2* ^*-/-*^ mice early after infection on day 3 suggest a critical role for Tpl2 in limiting virus replication during influenza virus infection.

### Tpl2 signaling in nonhematopoietic cells is necessary for limiting early virus replication

Airway epithelial cells are the primary targets for influenza virus infection. Early studies after the discovery of Tpl2 demonstrated high levels of Tpl2 expression in the lungs [21]. Moreover, similar to hematopoietic cells, Tpl2 regulation of signal transduction and cytokine gene induction was also demonstrated in airway epithelial cells [22]. To elucidate whether Tpl2 functions in hematopoietic or nonhematopoietic cells to limit virus replication, we assessed lung viral titers in chimeric mice in which WT or *Tpl2* ^*-/-*^ bone marrow cells were transferred into either WT or *Tpl2* ^*-/-*^ irradiated recipients. At day 3 pi, average lung viral titers were significantly higher in *Tpl2* ^*-/-*^ mice reconstituted with WT hematopoietic cells (Fig 1D). In contrast, there was no statistically significant increase in viral titers of WT mice that received *Tpl2* ^*-/-*^ bone marrow (Fig 1E). These data demonstrate that Tpl2 signaling within radioresistant, nonhematopoietic lung cells is necessary for limiting virus replication early after infection.

### Tpl2 is required for optimal IFNλ production during influenza infection *in vivo* and *in vitro*

**Fig 2:**
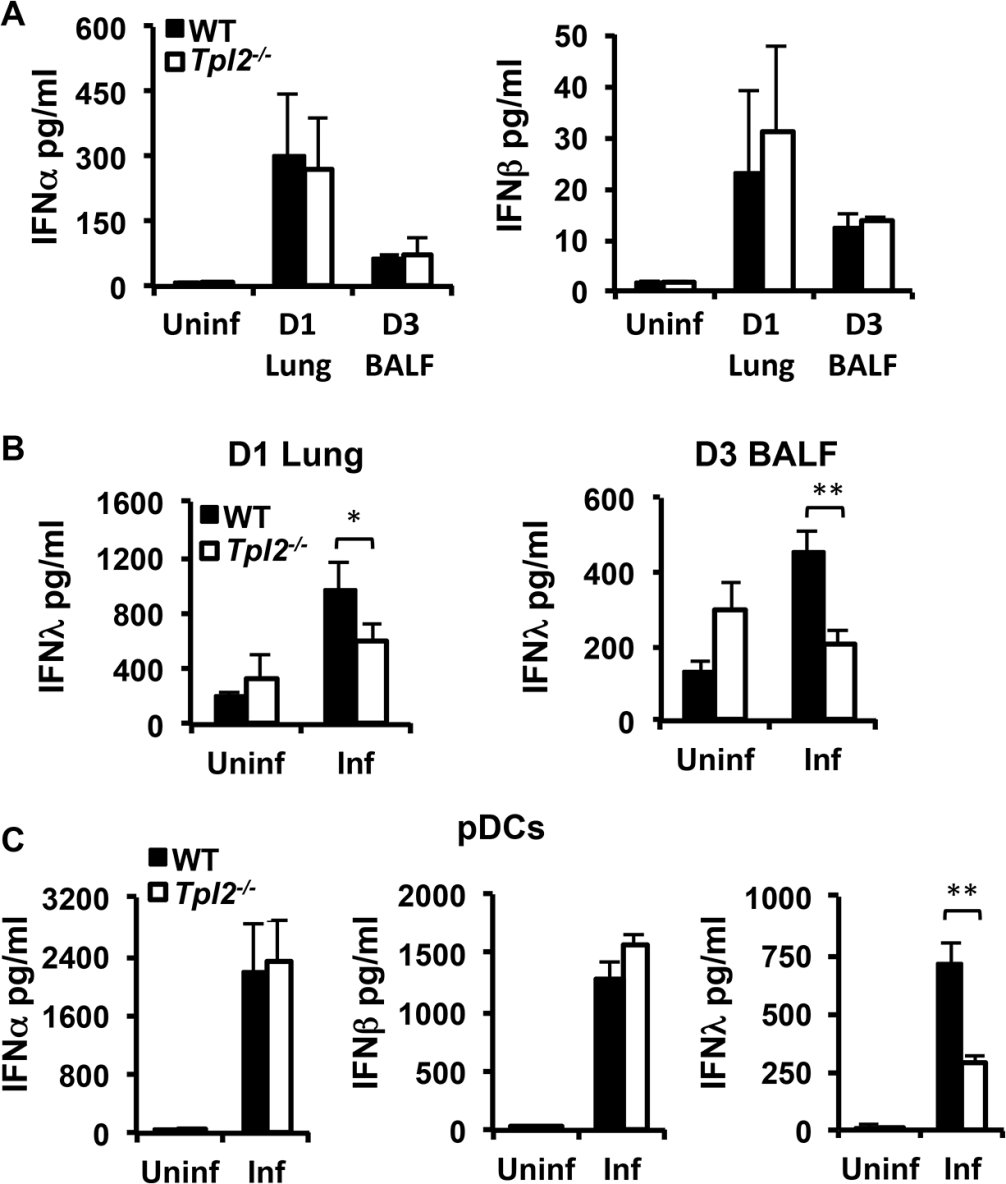
Tpl2 is required for optimal IFNλ production during influenza virus infection *in vitro* and *in vivo*. WT and *Tpl2* ^*-/-*^ mice were infected with 10^6^ pfu of X31 virus, and IFNα, β **(A)**, and λ **(B)** levels in D1 lung homogenates or D3 BALF were measured by ELISA or bead based assay. For IFNα, n = 4 uninfected and 7 infected mice per group; for IFNβ, n = 2 uninfected and 3 infected per group; for D1 IFNλ n = 5 (WT) and 6 (*Tpl2* ^*-/-*^) uninfected and 10 (WT) and 12 (*Tpl2* ^*-/-*^) infected; for D3 IFNλ n = 5 uninfected and 14 infected (WT) and 13 (*Tpl2* ^*-/-*^) infected. **(C)** pDCs from WT and *Tpl2* ^*-/-*^ mice were infected with WSN virus at an MOI of 0.2 for 24 hr, and IFNα, β and λ levels were measured. Data are representative of 3–4 independent experiments. * indicates *p*<0.05, ** indicates *p*<0.01.

Interferons are induced early during infection and are key factors initiating host protective antiviral responses [3]. To determine whether the observed increase in viral titers in *Tpl2* ^*-/-*^ mice is due to defective induction of IFNs, WT and *Tpl2* ^*-/-*^ mice were infected with 10^6^ pfu X31 virus, and IFNα/β/λ levels in lung homogenate or BALF were measured at day 1 or day 3 pi. Induction of both IFNα and β were comparable between WT and *Tpl2* ^*-/-*^ lung homogenates and BALF (Fig 2A). Notably, IFNλ secretion was significantly reduced in *Tpl2* ^*-/-*^ mice following influenza virus infection (Fig 2B). Surprisingly, while IFNλ was induced to a higher level compared to Type I IFNs in WT mice, there was minimal induction in *Tpl2* ^*-/-*^ mice in response to infection at both time points. Reduced IFNλ production in *Tpl2* ^*-/-*^ mice was independent of viral titers which were similar between WT and *Tpl2* ^*-/-*^ mice at day 1 pi (S2 Fig). Despite differences in IFNλ induction, total cellular infiltration and IFNγ levels in BALF were significantly elevated in *Tpl2* ^*-/-*^ mice compared to WT mice at day 3 pi (S3 Fig).

The observation that Tpl2 is uniquely required for IFNλ, but not IFNα or IFNβ, production in influenza-infected lungs is especially significant, because IFNλ is regarded as the principal IFN induced during influenza virus infection. Airway epithelial cells and pDCs are considered the major sources of IFNs during respiratory virus infections, including influenza [20, 23]. Although we observed a decrease in IFNλ levels in *Tpl2* ^*-/-*^ mice at day 1 pi, a more consistent and significant reduction was observed at day 3 pi, which corresponds to the migration of pDCs to infected lungs [23]. Since Tpl2 is required for macrophage and neutrophil migration during acute inflammation [9, 24], we investigated whether Tpl2 similarly regulates the recruitment of pDCs to the infected lung. The reduction in IFNλ levels in influenza-infected *Tpl2* ^*-/-*^ mice was not due to impaired recruitment of pDCs (S4 Fig). To investigate whether defective IFN induction by pDCs contributes to the reduced IFNλ in BALF from *Tpl2* ^*-/-*^ mice during influenza infection, bone marrow-derived pDCs (CD11c^+^B220^+^CD11b^-^) from WT and *Tpl2* ^*-/-*^ mice were infected with influenza virus A/WSN/1933 (H1N1), and the production of IFNα, β and λ was assessed. Consistent with *in vivo* infections, the levels of both IFNα and IFNβ were comparable between WT and *Tpl2* ^*-/-*^ cells, whereas IFNλ secretion was significantly less in *Tpl2* ^*-/-*^ pDCs infected with influenza virus (Fig 2C). A similar reduction in IFNλ induction was also observed in Tpl2-deficient cells infected with X31 influenza virus strain (S5 Fig). Collectively, these data demonstrate the unique requirement for Tpl2 in IFNλ production during influenza infection *in vitro* and *in vivo*.

### Tpl2 differentially regulates IFN production in response to model viral ligands in a cell type-specific manner

**Fig 3:**
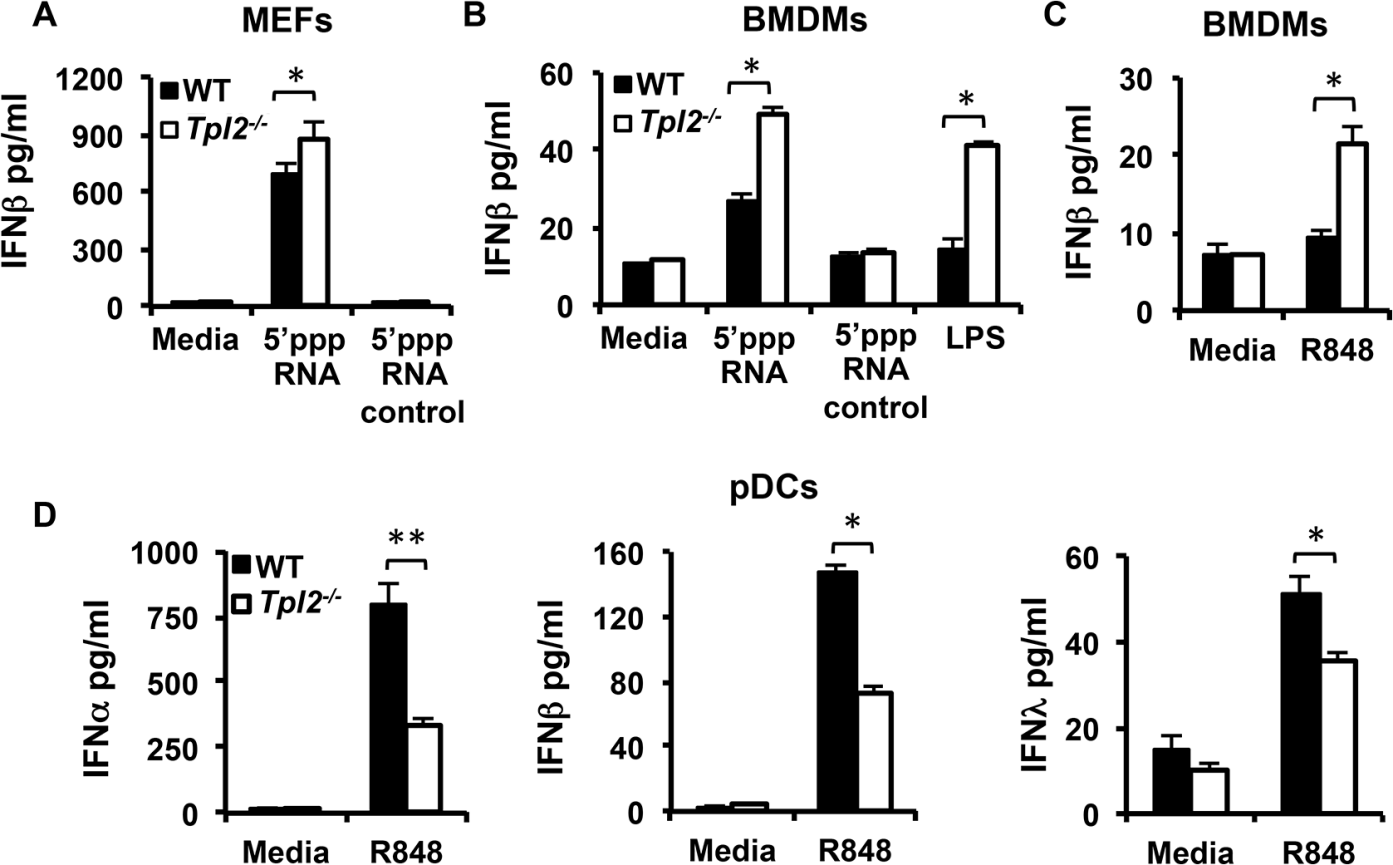
Tpl2 differentially regulates IFN production in response to model viral ligands in a cell type-specific manner. MEFs **(A)** or BMDMs **(B-C)** from WT and *Tpl2* ^*-/-*^ mice were transfected with 5’ppp-RNA or stimulated R848 or LPS for 24 hr, and IFNβ levels were measured by ELISA. **(D)** Plasmacytoid DCs were stimulated with R848 for 24 hr, and IFNα, β and λ levels were measured. Data are representative of 3–4 independent experiments. Graphs show means±SD. * indicates *p*<0.05, ** indicates *p*<0.01.

During influenza virus infection, receptors from both TLR and RLR families recognize viral PAMPs and trigger rapid induction of IFNs. Recognition of viral components by PRRs typically occurs in respiratory epithelial cells, alveolar macrophages, DCs and pDCs in a cell type-specific manner [25]. The major receptors involved in recognition of influenza virus are TLR7, which recognizes single-stranded viral RNA, and RIG-I, which recognizes the 5’-triphosphate of single-stranded RNA genomes (5’ppp-RNA). The single-stranded RNA genome is recognized through endosomal TLR7 in pDCs [18] in contrast to epithelial cells and DCs where virus recognition is mediated primarily by the cytosolic sensor RIG-I [26]. We therefore investigated whether differential regulation of IFN production observed during infection is due to differences in Tpl2-mediated sensing by PRRs. MEFs and bone marrow-derived macrophages (BMDMs) from WT and *Tpl2* ^*-/-*^ mice were either transfected with the RIG-I ligand 5’ppp-RNA or stimulated with the TLR7 ligand R848 [27], and IFNβ production was measured by ELISA. Consistent with previous studies using the TLR4 ligand LPS [4], IFNβ production was significantly increased in *Tpl2* ^*-/-*^ cells treated with both 5’ppp-RNA and R848 (Fig 3A–3C). This increase in IFNβ correlated with impaired ERK phosphorylation in Tpl2-deficient cells in response to these ligands (S6 Fig). Unlike epithelial cells and DCs, virus recognition in pDCs is mediated via TLRs rather than RLHs, and Type I IFN production occurred normally in RIG-I-deficient pDCs infected with RNA viruses [18, 26]. To determine whether Tpl2 regulates TLR7-mediated IFN production by pDCs, bone marrow-derived pDCs from WT and *Tpl2* ^*-/-*^ mice were treated with the TLR7 ligand, R848, and IFN levels were quantitated. Consistent with previous studies using the TLR9 ligand CpG [4], and in contrast to BMDMs, secretion of both IFNα and IFNβ were significantly decreased in culture supernatants from *Tpl2* ^*-/-*^ pDCs treated with R848 (Fig 3D). Notably, IFNλ secretion was also significantly less in *Tpl2* ^*-/-*^ pDCs compared to WT cells in response to R848 (Fig 3D). Unlike *Ifna* but similar to NFκB-regulated *Il12p40* and *Tnfa* [28], IFNλ3 *(Il28b)* transcription occurred early, by 2 hr of stimulation (S7 Fig). Collectively, these data demonstrate that Tpl2 differentially regulates IFN production downstream of PRRs involved in influenza virus sensing in a cell type-specific manner.

### ERK and Akt are involved in Tpl2-dependent IFNλ production in pDCs

**Fig 4:**
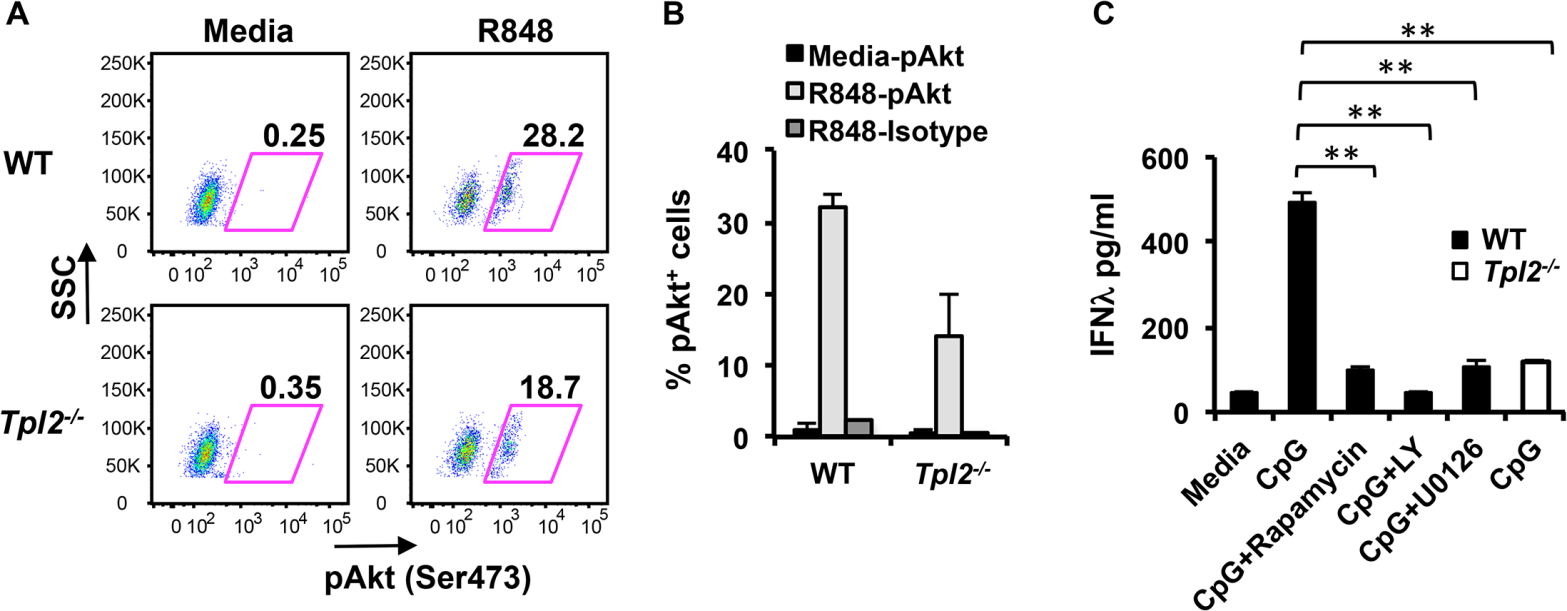
ERK and Akt are involved in Tpl2-dependent IFNλ production in pDCs. pDCs from WT and *Tpl2* ^*-/-*^ mice were stimulated with R848 for 18 hr, and analyzed by intracellular staining for pAkt^Ser473^. **(A)** Representative flow cytometry plots showing pAkt^Ser473^ staining within pDCs. **(B)** Proportion of pAkt positive pDCs from 2 independent experiments. **(C)** pDCs were pretreated with inhibitors for 30 min before stimulation with CpG, and IFNλ levels were measured by ELISA. Data are representative of 2 (A-B) or 3 (C) independent experiments. Graphs show means±SD. * indicates *p*<0.05, ** indicates *p*<0.01.

The importance of IFNλs in host protection against many viruses is well established, however, the mechanisms that regulate their production are largely unexplored. Common mechanisms have been postulated to regulate Type I and III IFNs during viral infections [29, 30]. Despite their importance in mediating Type I IFN production in pDCs [4, 31], the significance of MAP kinase and PI3 kinase cascades in murine IFNλ production has not been directly investigated. In order to elucidate the potential mechanism by which Tpl2 regulates IFNλ production in pDCs, we evaluated the involvement of ERK and PI3K-mTOR signaling in IFNλ induction. Tpl2 regulation of both ERK and mTOR-Akt signaling in different cell types has been reported previously [8, 32–34]. In addition to the MEK/ERK pathway [4], we demonstrate that Tpl2 also promotes mTOR/Akt signaling in pDCs as determined by a decrease in the proportion of phospho-Akt^+^ pDCs in the absence of Tpl2 signaling (Fig 4A and 4B). To confirm whether ERK, PI3K or mTOR signaling also contributes to IFNλ production in pDCs, cells were pre-treated with rapamycin (mTOR inhibitor), LY294002 (PI3K inhibitor) or U0126 (MEK inhibitor) 30 min prior to TLR stimulation, and CpG-induced IFNλ secretion was measured by ELISA. CpG was used as the stimulant in these experiments because TLR9 ligation induced higher levels of IFNλ compared to TLR7 stimulation with R848. Pharmacological inhibition of each of these signaling pathways significantly reduced IFNλ secretion to the levels observed in *Tpl2* ^*-/-*^ cells (Fig 4C). In contrast, only a modest reduction in IFNλ induction was observed in Tpl2-deficient cells treated with rapamycin or U0126 (S8 Fig). These results demonstrate the significance of Tpl2 and both MAPK and PI3 kinase signaling cascades in regulating IFNλ production in pDCs.

### Induction of IFNλ in influenza virus-infected lungs occurs independently of Type I IFN signaling

**Fig 5:**
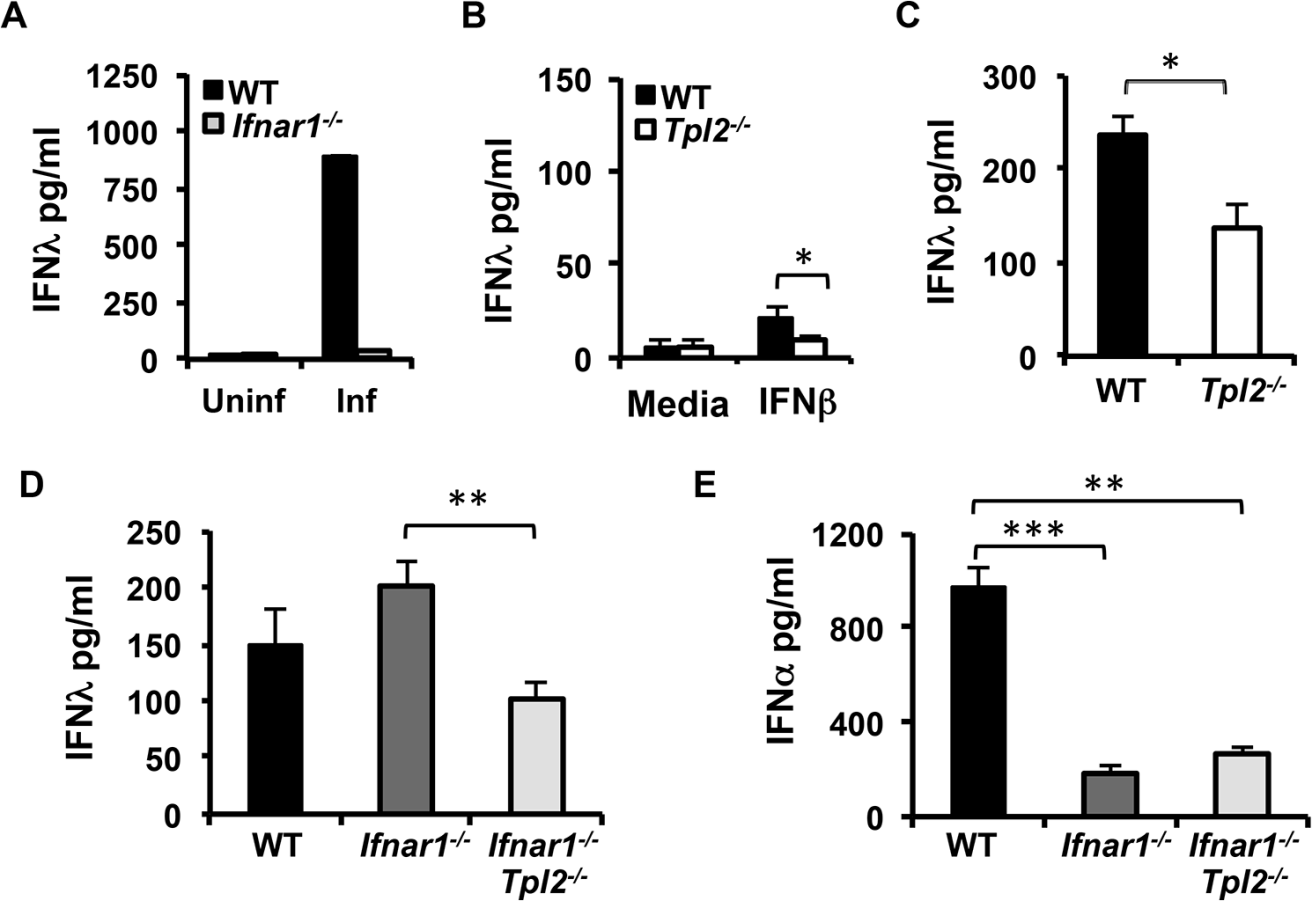
IFNλ production is IFNAR-independent in influenza virus-infected lungs. **(A)** Flt3 ligand-derived DCs from WT and *Ifnar1* ^*-/-*^ mice were infected with WSN virus for 24 hr, and IFNλ secretion was measured by ELISA. **(B)** Flt3 ligand-derived DCs from WT and *Tpl2* ^*-/-*^ mice were treated with IFNβ for 24 hr, and IFNλ secretion was quantitated by ELISA. Data are representative of 2 **(A)** or 3 **(B)** independent experiments. Graphs show means±SD. **(C)** WT and *Tpl2* ^*-/-*^ mice were infected with 10^4^ pfu of X31 virus, and IFNλ levels in lung homogenates were measured by ELISA on D3 pi; n = 5 WT and 5 *Tpl2* ^*-/-*^ mice. WT, *Ifnar1* ^*-/-*^ and *Ifnar1* ^*-/-*^ *Tpl2* ^*-/-*^ mice were infected with 10^4^ pfu of X31 virus, and IFNλ **(D)** and IFNα **(E)** levels in lung homogenates were measured by ELISA on D3 pi; n = 4 WT, 5 *Ifnar1* ^*-/-*^ and 9 *Ifnar1* ^*-/-*^ *Tpl2* ^*-/-*^ mice. Graphs show means±SEM. * indicates *p*<0.05, ** indicates *p*<0.01, *** indicates *p*<0.001.

Robust production of Type I IFNs in pDCs is dependent upon IRF7 and autocrine IFN signaling, and consequently IFNα secretion is abrogated in both *Irf7* ^*-/-*^ and *Ifnar1* ^*-/-*^ pDCs [35]. Similar to IFNα, and as reported previously [20], IFNλ production was abolished in *Ifnar1* ^*-/-*^ pDCs infected with influenza virus (Fig 5A) demonstrating the absolute requirement for IFNAR signaling in IFNλ secretion by pDCs. Induction of IFNλ in response to direct IFN stimulation has been reported in hepatocyte carcinoma HepG2 cell lines [36]. Although a high dose of IFNβ could induce modest IFNλ secretion, the levels induced were lower than that induced by TLR-stimulation, demonstrating that IFN/IRF7 signaling alone is not sufficient for driving high levels of IFNλ secretion (Fig 5B). Nevertheless, Tpl2 contributed to IFNAR-induced IFNλ production, since significantly less IFNλ was secreted by *Tpl2* ^*-/-*^ pDCs directly treated with IFNβ (Fig 5B). In addition to demonstrating the role of Tpl2 in IFNAR-mediated IFNλ production, these data also suggest a role for Tpl2 in directly transducing Type I IFN signals.

To determine whether Tpl2 regulates IFNλ production in influenza virus-infected lungs directly via virus sensing pathways or indirectly via IFNAR feedback signaling, we assessed IFNλ levels in lung homogenates from mice that are deficient in both Tpl2 and IFNAR1. Consistent with reduced IFNλ levels in BALF from *Tpl2* ^*-/-*^ mice day 3 pi (Fig 2B), IFNλ levels were similarly reduced in day 3 lung homogenates (Fig 5C). IFNλ levels were significantly decreased in *Ifnar1* ^*-/-*^ *Tpl2* ^*-/-*^ compared to *Ifnar1* ^*-/-*^ mice, demonstrating that Tpl2 promotes early IFNλ induction independent of Type I IFN signaling (Fig 5D). Notably, the level of IFNλ induction was similar in *Tpl2* ^*-/-*^ and *Ifnar1* ^*-/-*^ *Tpl2* ^*-/-*^ mice (Fig 5C and 5D). In striking contrast to the abrogation of IFNλ production in *Ifnar1* ^*-/-*^ pDCs (Fig 5A), IFNλ production occurred normally in *Ifnar1* ^*-/-*^ mice (Fig 5D). Consistent with the critical role of IFNAR signaling in IFNα induction, IFNα levels were significantly diminished in both *Ifnar1* ^*-/-*^ and *Ifnar1* ^*-/-*^ *Tpl2* ^*-/-*^ mice (Fig 5E). These data demonstrate that Tpl2-dependent IFNλ production during influenza virus infection is IFNAR-independent.

### Tpl2 mediates IFN signaling and induction of IFN-stimulated genes (ISGs)

**Fig 6:**
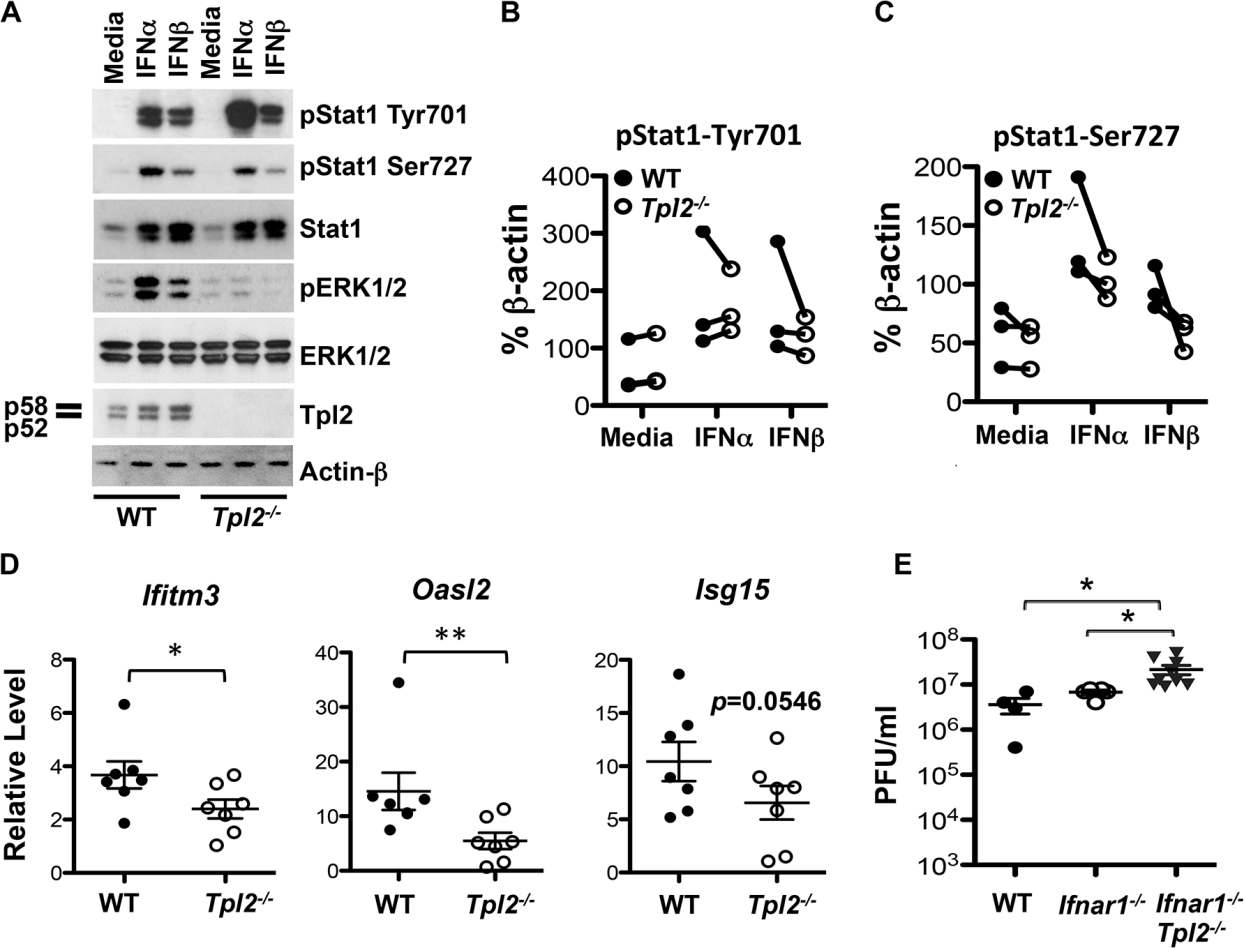
Tpl2 mediates IFN signaling and induction of ISGs. **(A)** BMDMs from WT and *Tpl2* ^*-/-*^ mice were stimulated with IFNα or IFNβ for 1 hr, and STAT1 and ERK phosphorylation were assessed by immunoblotting. Data are representative of 3 independent experiments. Average intensities of pSTAT1^Tyr701^ **(B)** and pSTAT1^Ser727^ **(C)** bands normalized to actin bands by densitometric analysis. Data collected on the same day are connected by lines. **(D)** WT and *Tpl2* ^*-/-*^ mice were infected with 10^6^ pfu of X31 virus, and the expression of *Ifitm3*, *Isg15* and *Oasl2* in lung tissue D1 pi was measured by RT-PCR with normalization to actin mRNA and WT uninfected sample (n = 7). **(E)** WT, *Ifnar1* ^*-/-*^ and *Ifnar1* ^*-/-*^ *Tpl2* ^*-/-*^ mice were intranasally infected with 10^4^ pfu of X31 virus, and lung viral titers on D3 pi were enumerated by plaque assays; n = 4 WT, 5 *Ifnar1* ^*-/-*^ and 9 *Ifnar1* ^*-/-*^ *Tpl2* ^*-/-*^ mice. * indicates *p*<0.05, ** indicates *p*<0.01.

Both IFNα/β and IFNλ are known to induce expression of ISGs that establish an antiviral state in infected tissue to prevent virus replication and spread [3, 37]. Because of the observed increase in early virus replication in *Tpl2* ^*-/-*^ mice (Fig 1A), we questioned whether Tpl2 regulates the induction of ISGs. We first addressed whether Tpl2 regulates IFN signaling. BMDMs from WT and *Tpl2* ^*-/-*^ mice were stimulated with IFNα or IFNβ, and activation of downstream cascades, especially STAT1, which is the principle regulator of IFN responses, was evaluated by immunoblotting. BMDMs were used in these experiments due to limited availability of pDCs. The phosphorylation of STAT1 Tyr701 and Ser727, which is necessary for maximal STAT1 transcriptional activation, were examined [38]. While phosphorylation of Tyr701 occurred normally in Tpl2-deficient cells in response to stimulation with Type I IFNs, a consistent reduction in Ser727 phosphorylation was observed in *Tpl2* ^*-/-*^ cells compared to WT cells (Fig 6A–6C). In addition to the classical JAK-STAT pathway, signaling via the Type I IFN receptor also activates other downstream cascades including MAP kinases [39]. Despite the existence of multiple MAP3 kinases, Tpl2 has an essential, non-redundant role in transducing ERK activation signals during TLR, TNF- and IL-1-receptor signaling [7, 8]. We therefore investigated whether Tpl2 is similarly required for ERK activation during Type I IFN signaling, or whether other MAP3Ks could fulfill this role. ERK phosphorylation was strongly induced by both IFNα and IFNβ. Importantly, ERK phosphorylation was absent in *Tpl2* ^*-/-*^ BMDMs stimulated with IFNα/β demonstrating an absolute requirement for Tpl2 in transducing ERK activation signals in response to Type I IFNs (Fig 6A). Of note, unlike LPS- and TNFα-treated BMDMs and similar to poly I:C-, CpG-, and IL-1β-treated BMDMs [17, 40], no mobility shift (indicative of phosphorylation) or degradation of the p58 isoform of Tpl2 was detected following stimulation with Type I IFNs (Fig 6A). Consistent with our previous studies [41], both Tpl2 protein and mRNA expression were induced upon either Type I IFN stimulation or influenza virus infection (Fig 6A and S9 Fig). Overall, these data demonstrate that Tpl2 contributes to Type I IFN signaling.

Since Tpl2 is known to modulate the antiviral transcriptome [16], we next investigated whether the induction of ISGs in infected lungs is impaired in the absence of Tpl2. The induction of *Ifitm3*, *Isg15* and *Oasl2*, ISGs known to be important in limiting influenza virus infection [25], were measured. We observed a modest, but statistically significant decrease in *Ifitm3* and *Oasl2* expression in *Tpl2* ^*-/-*^ compared to WT mice infected with influenza virus (Fig 6D). A trend towards reduction in *Isg15* expression was also noted in *Tpl2* ^*-/-*^ mice (Fig 6D). In addition to infected lungs, the induction of *Oasl2* was significantly reduced in *Tpl2* ^*-/-*^ BMDMs, while the expression of *Ifitm3* and *Isg15* was largely unaffected by Tpl2 ablation (S10 Fig). These data demonstrate that Tpl2 promotes the induction of ISGs in influenza-infected lungs to limit virus replication.

Although Tpl2 is important in transducing Type I IFN signals, this would not alone account for the increase in viral titers or reduction in ISGs observed in *Tpl2* ^*-/-*^ mice, since either Type I or Type III IFN is sufficient for protection during influenza virus infection. This is because both types of IFNs drive redundant amplification loops inducing the expression of similar antiviral genes [42]. To investigate whether IFNAR signaling contributes to the observed increase in virus replication, we next assessed lung viral titers in mice deficient in both Tpl2 and IFNAR1. Consistent with previous studies [20], viral titers were comparable between WT and *Ifnar1* ^*-/-*^ mice (Fig 6E). Although average lung viral titers were significantly higher in *Ifnar1* ^*-/-*^ *Tpl2* ^*-/-*^ mice compared to both WT and *Ifnar1* ^*-/-*^ mice (Fig 6E), the titers were similar to those observed in *Tpl2* ^*-/-*^ mice (Fig 1A). These data demonstrate that Tpl2 restricts early virus replication in an IFNAR-independent manner.

### Tpl2 ablation limits the induction of antigen-specific CD8^+^ T cells and enhances susceptibility to influenza infection

**Fig 7:**
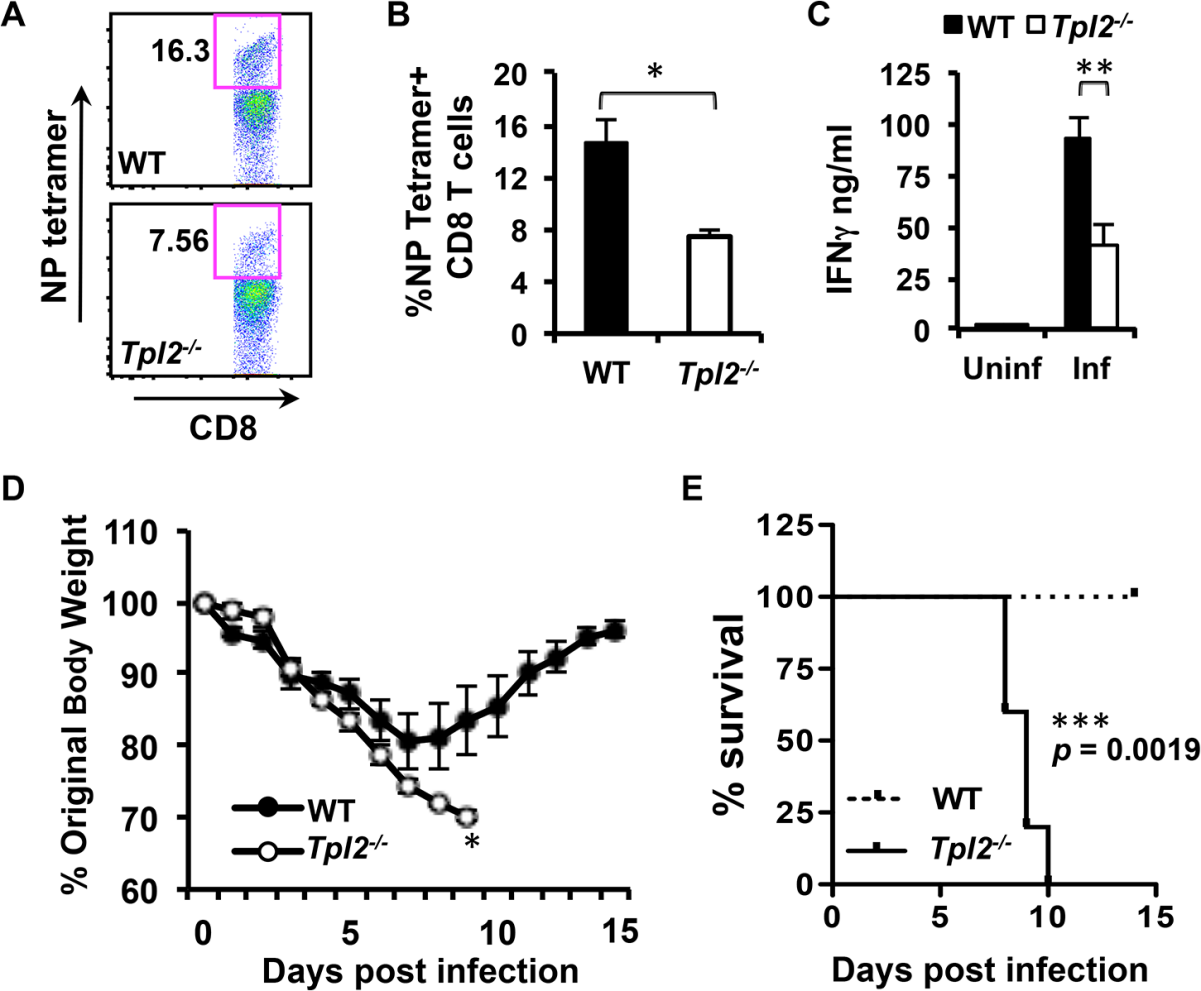
Tpl2 ablation limits antigen-specific CD8^+^ T cell responses and enhances susceptibility to influenza infection. **(A-B)** WT and *Tpl2* ^*-/-*^ mice were infected with 10^4^ pfu of X31 virus, and the proportion of NP_366–374_ tetramer positive CD8^+^ T cells in BAL were assessed. **(C)** BAL cells were stimulated with a cocktail of influenza immunodominant peptides for 24 hr, and secretion of IFNγ was measured by ELISA; n = 5. **(D-E)** WT and *Tpl2* ^*-/-*^ mice were infected with 10^4^ pfu of X31 virus, body weights were recorded daily for 14 days, and mice exhibiting severe signs of disease, including more than 30% weight loss were euthanized. Data are representative of 3 independent experiments; n = 5. * indicates *p*<0.05, ** indicates *p*<0.01, *** indicates *p*<0.005.

Even though the observed reduction in ISGs helps to explain the early increase in viral titers, a more pronounced and biologically significant increase in viral titers was observed at day 7 pi which correlates with the recruitment of influenza-specific CD8^+^ T cells to the lungs [43]. Since many seminal studies have identified CD8^+^ T cells as the major mediators of influenza virus clearance from infected lungs [44, 45], we investigated whether virus-specific CD8^+^ T cell responses are impaired in *Tpl2* ^*-/-*^ mice. Consistent with defective viral clearance observed in *Tpl2* ^*-/-*^ mice, induction of protective nucleoprotein (NP)-specific CD8^+^ T cells [46] was significantly reduced in BAL cells from *Tpl2* ^*-/-*^ mice compared to WT animals (Fig 7A and 7B). In addition, antigen-specific secretion of IFNγ was also decreased in BAL cells from *Tpl2* ^*-/-*^ mice (Fig 7C). During the course of this experiment, we unexpectedly observed severe clinical signs in *Tpl2* ^*-/-*^ mice despite the fact that the mice were infected with the low pathogenicity A/HK-X31(H3N2) (X31) influenza virus. To confirm whether Tpl2 ablation alters the susceptibility to influenza virus infection, WT and *Tpl2* ^*-/-*^ mice were infected with 10^4^ pfu of X31 virus, and weight loss and clinical symptoms were monitored over a period of 14 days. All *Tpl2* ^*-/-*^ mice exhibited severe clinical signs and succumbed to infection by day 10 pi, whereas all WT animals survived and returned to pre-infection body weights by day 14 pi (Fig 7D and 7E). Similar to infection with X31 virus, *Tpl2* ^*-/-*^ mice infected with the virulent PR8 [A/Puerto Rico/8/34 (PR8; H1N1)] strain showed increased disease severity compared to WT mice, although not to the same extent seen with the low pathogenicity virus (S11 Fig). Body weights were collected to day 10 pi, at which time the *Tpl2* ^*-/-*^ mice met the humane endpoints of the study. At this time point, the body weights were just beginning to show the characteristic switch between the WT and *Tpl2* ^*-/-*^ mice, such that the *Tpl2* ^*-/-*^ mice were showing more severe clinical signs of disease. Accordingly, systemic pro-inflammatory cytokine levels were also increased in the *Tpl2* ^*-/-*^ mice at day 10 pi. Analysis of BAL cells also showed decreased antigen-specific CD8^+^ T cell responses in *Tpl2* ^*-/-*^ mice compared to WT mice at this late time point, consistent with the observations with X31 infections. Collectively, these data demonstrate the critical role of Tpl2 in promoting viral clearance and restricting morbidity and mortality associated with influenza virus infection.

## Discussion

**Fig 8:**
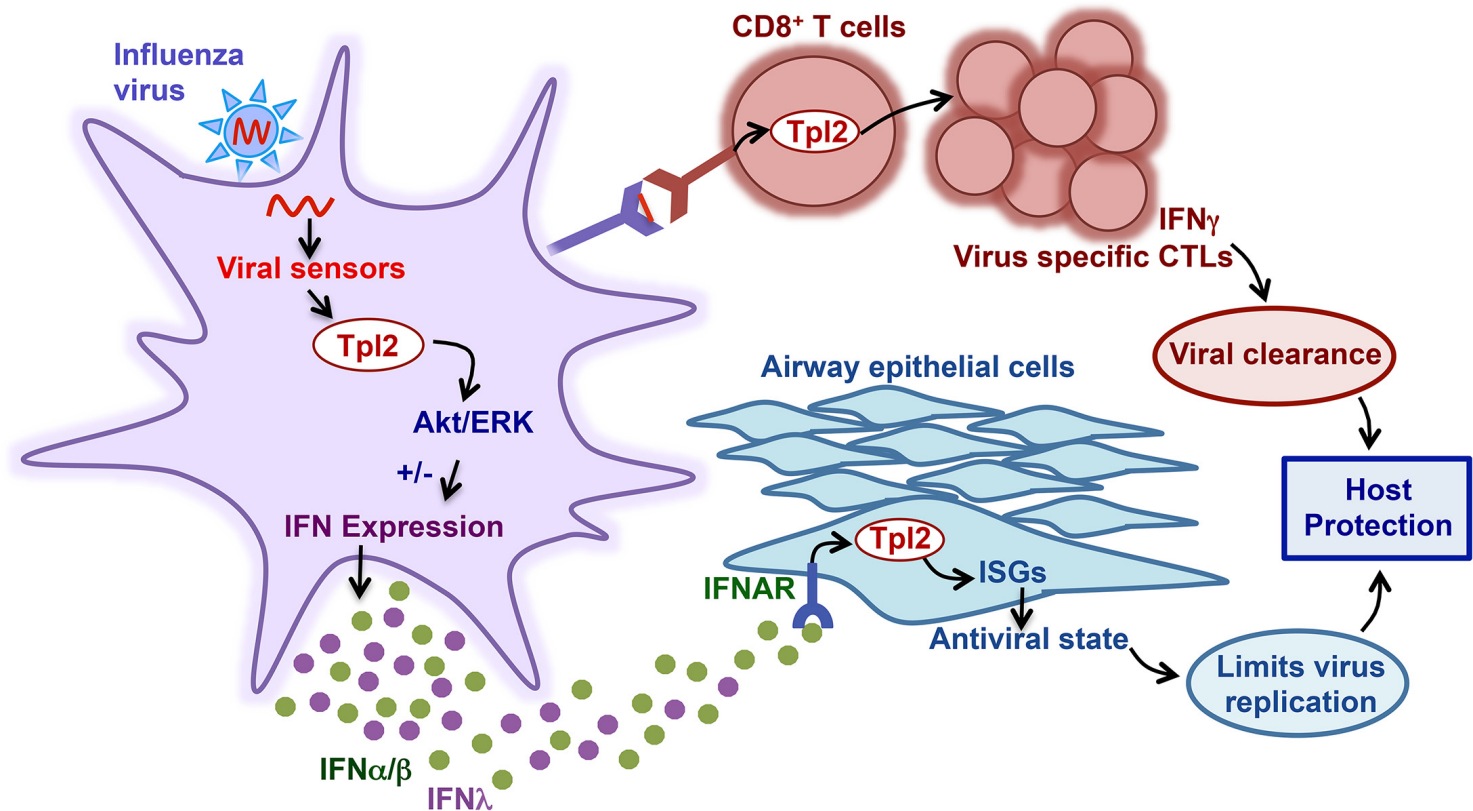
Model of Tpl2 regulation of antiviral immune responses. During influenza virus infection, the viral RNA is recognized by TLR7 (in pDCs) or RIG-I (in other cell types). Virus recognition by these receptors activates various downstream signaling cascades, including Tpl2-ERK signaling, which either positively or negatively regulates secretion of IFNα/β or IFNλ in a cell-type specific manner. Specifically, Tpl2 inhibits TLR- and RLR-induced IFNα/β production in macrophages, but promotes IFNα/β and IFNλ in TLR-stimulated pDCs. Tpl2 is also involved in transducing Type I IFN signals. Moreover, Tpl2 regulates induction of ISGs, which are important in limiting virus replication. In addition to early innate responses, Tpl2 promotes expansion of virus-specific CD8^+^ T cells that facilitate viral clearance from infected lungs. Therefore, by integrating both innate and adaptive antiviral responses, Tpl2 promotes host protection during influenza virus infection.

Tpl2 is now appreciated to regulate the induction of Type I and Type II IFNs as well as other cytokines that may contribute to antiviral responses. However, there is very limited information on how Tpl2 coordinates antiviral immune responses *in vivo*. In this study, we demonstrate Tpl2’s obligate role in promoting antiviral responses and viral clearance during influenza virus infection. These findings are important because influenza virus is a ubiquitous seasonal virus that afflicts millions of people annually, causing significant morbidity, mortality and socio-economic burdens [47]. Therefore, understanding the role of host factors like Tpl2 in restricting morbidity and mortality associated with influenza virus infection is critical for developing disease intervention strategies. Mechanistically, Tpl2 promotes the induction of ISGs and virus-specific CD8^+^ T cells that facilitate viral clearance as shown in the proposed model (Fig 8). Thus, the findings reported here establish an essential role for Tpl2 in host protective innate and adaptive antiviral responses.

Tpl2 deficiency led to cell-type specific alterations in the regulation of Type I IFN production. Specifically, IFNβ production was increased in response to LPS, R848 and the RIG-I ligand, 5’-triphosphate RNA in *Tpl2* ^*-/-*^ MEFs and BMDMs. In contrast, Type I IFN was significantly reduced in pDCs in response to TLR7 stimulation with R848. This differential regulation of Type I IFN production by Tpl2 in different cell types in response to TLR ligands is consistent with a previous report by O’Garra and colleagues [4]. Importantly, we also demonstrated that Tpl2 similarly functions as a negative regulator of Type I IFN production upon activation of the RIG-I cytosolic sensor with 5’-triphosphate RNA.

One striking observation was the absolute requirement for Tpl2 in the TLR-dependent induction of both Type I (IFNα/β) and Type III IFNs (IFNλ) by pDCs. The fact that pDCs uniquely require Tpl2 for production of both Type I and Type III IFNs suggests that pDCs differ fundamentally from BMDMs and MEFs in their signaling pathways. Indeed, impaired IFN production correlated with reduced activation of the PI3K/Akt signaling pathway in *Tpl2* ^*-/-*^ pDCs. This finding is also consistent with the observation that the PI3K/Akt pathway appears to be especially important in driving TLR-dependent IFN expression by pDCs [31].

In addition to cell-type specific regulation, Tpl2 also differentially regulates the production of Type I and Type III IFNs during viral infection. Importantly, influenza virus has been reported to utilize the Raf pathway to activate ERK, which explains why Type I IFN induction occurs in a Tpl2-independent manner in mice and pDCs infected with influenza virus [48]. On the contrary, IFNλ production was uniquely dependent upon Tpl2 during the course of influenza infection *in vitro* and *in vivo*. Although Type I and Type III IFNs have common regulatory elements in their promoters and are usually co-expressed in response to viruses and TLR ligands [36], selective induction of IFNλ by transcription factors NFκB and IRF1 has been reported [49, 50]. The distinct requirement for Tpl2 in IFNλ induction in virus-infected pDCs likely represents the unique requirement of the IFNλ promoter for an early NFκB-dependent priming event. In support of this, our own data demonstrate that IFNλ induction is rapid and parallels the regulation of NFκB-dependent genes more closely than IFNα (S7 Fig).

With the exception of a recent study reporting that p38, but not ERK, is required for *Ifnl1* expression in human cells [49], the roles of MAPK or PI3K pathways in the regulation of IFNλs have not been evaluated. Although the regulation of IFNλ1 by PI3K-mTOR is still unexplored, our data demonstrate a different mechanism of IFNλ3 regulation that relies on the Tpl2-ERK pathway in contrast to the p38-dependent regulation described for IFNλ1. Therefore, in addition to transcription factors [30], diverse signaling cascades also specify induction of different IFNs.

The complexity of the IFN response is not completely understood, since multiple signaling cascades and transcription factors activated during IFN signaling can independently or cooperatively regulate the transcriptional response to IFNs [39]. Importantly, our data demonstrate the involvement of Tpl2 in IFN signaling leading to the phosphorylation of ERK and STAT1^Ser727^. Previous studies have demonstrated the significance of STAT1^Ser727^ phosphorylation for full transcriptional activation and induction of ISGs [38, 51]. Conflicting reports exist regarding the identity of the serine kinase responsible for STAT1^Ser727^ phosphorylation; different kinases including p38, ERK and PKC-δ have been implicated [52–54]. Importantly, an association of ERK with STAT1 and a requirement of ERK activity for expression of ISGs have been demonstrated [55]. Tpl2 regulation of STAT1^Ser727^ phosphorylation and induction of ISGs might be indirect via its regulation of ERK phosphorylation during IFN signaling. In addition to regulating ISG transcription, Tpl2-ERK signaling also regulates the phosphorylation and dissociation of the translation initiation factor 4E-Bp-eIF4E complex, which is involved in cap-dependent translation of many genes, including ISG15 [34, 56]. Therefore, the Tpl2-ERK pathway regulates the biological effects of IFNs at the transcriptional level and possibly also at the posttranscriptional level.

Although MAP kinase pathways are known to be activated in response to IFNs, the importance of Tpl2 in regulating IFN-inducible effectors has not yet been described. The induction of ISGs is mainly attributed to IFN-stimulated gene factor-3 (ISGF3; consists of STAT1, STAT2 and IRF9). In addition to ISGF3, IRF7 can also act independently to regulate transcription of antiviral genes, and Tpl2 has been shown to promote IRF7-dependent transcription [16]. However, normal induction of IFNα/β during influenza virus infection argues against a major role for IRF7 in the observed phenotype, since IRF7 is regarded as the ‘master regulator’ of Type I IFN induction [35].

To understand the mechanism by which Tpl2 exerts its antiviral effect, we examined the contribution of Tpl2 to virus replication in different cellular compartments and in the context of IFNAR deficiency. Using bone marrow chimeras, we demonstrated that Tpl2 was required within the nonhematopoietic compartment to restrict early virus replication. This likely reflects Tpl2 functions in airway epithelial cells, the primary target of influenza virus. In this regard, Tpl2 is known to be expressed and to regulate inflammation within airway epithelial cells [22]. Studies using *Ifnar1* ^*-/-*^ *Il28ra* ^*-/-*^ mice have also demonstrated that interferon responsiveness of these cells is critical for restricting early viral replication [42]. It is well known that abrogation of Type I IFN signaling does not increase influenza virus replication due to the presence of compensatory Type III IFNs [57]. Consistent with this, we observed that Tpl2 ablation promoted virus replication to the same extent on both *Ifnar1* ^*+/+*^ and *Ifnar1* ^*-/-*^ genetic backgrounds. The 50% reduction in IFNλ levels that we observed in *Tpl2* ^*-/-*^ mice on day 3 pi is unable to explain the increase in virus replication, because compensatory Type I IFNs are induced to normal levels. Furthermore, the presence of IFNs, rather than quantity, is important in driving antiviral responses [42]. One possible explanation for the increased viral replication in *Tpl2* ^*-/-*^ mice is that Type III IFN signaling is also Tpl2-dependent, like we have demonstrated for Type I IFNs. Additional studies using *Il28ra* ^*-/-*^ mice are needed to determine the contribution of Tpl2 to Type III IFN signaling.

In addition to antiviral innate responses, we also identified a critical role for Tpl2 in the induction of antigen-specific CD8^+^ T cell responses. This is in contrast to a recent study reporting a major role for Tpl2 in human, but not murine, CD8^+^ T cell responses [58]. The impaired induction of virus-specific CD8^+^ T cells resulting in defective viral clearance and increased mortality in *Tpl2* ^*-/-*^ mice clearly warrants detailed studies on Tpl2 regulation of effector CD8^+^ T cell responses.

The increased mortality observed in *Tpl2* ^*-/-*^ mice infected with X31 virus was surprising because infection with this low pathogenicity virus does not typically cause severe clinical signs or mortality in mice. Even though IFNλ production was impaired in *Tpl2* ^*-/-*^ mice, this defect is not sufficient to explain their increased morbidity and mortality, because several studies have shown that *either* Type I or Type III IFN alone is sufficient to limit influenza virus infection [20, 42, 59]. In addition to impaired CD8^+^ T cell responses [45], the reduction in expression of some ISGs may also contribute to the enhanced pathogenesis, since defective expression of individual antiviral factors, like IFITM3, can alter the course of infection [60]. Early increases in virus replication in Tpl2-deficient lung stromal cells, demonstrated by bone marrow chimera experiments, coupled with defective viral clearance by CD8^+^ T cells likely potentiate the inflammatory response, which is considered a major factor contributing to morbidity and mortality during pathogenic influenza infection [61].

Overall, our study establishes Tpl2 as a host factor with intrinsic ability to restrict influenza virus replication and also demonstrates immune regulatory functions of Tpl2 within the lungs. The involvement of Tpl2 in major virus sensing pathways as well as antiviral signaling cascades suggests a key role for Tpl2 in integrating antiviral responses. These results are especially significant considering a very recent study demonstrating the requirement of IRF7 as well as Type I and Type III IFNs, all regulated by Tpl2, in protecting humans from life-threatening influenza virus infection [62]. Whether Tpl2 similarly restricts the replication of other classes of viruses requires further investigation. The findings reported here also suggest that therapeutic inhibition of Tpl2 during chronic inflammatory diseases might predispose patients to viral infections.

## Materials and Methods

### Ethics statement

All animal experiments were performed in accordance to the national guidelines provided by “The Guide for Care and Use of Laboratory Animals” and The University of Georgia Institutional Animal Care and Use Committee (IACUC). The Institutional Animal Care and Use Committee (IACUC) of the University of Georgia approved all animal experiments (Assurance Number A3437-01).

### Mice and viruses

Wild type (WT) C57BL/6J (CD45.2^+^) mice were purchased from The Jackson Laboratory. *Tpl2* ^*-/-*^ mice backcrossed to C57B6/J were kindly provided by Dr. Philip Tsichlis (Tufts University) and Thomas Jefferson University. For some experiments, littermate control WT and *Tpl2* ^*-/-*^ mice were obtained by interbreeding *Tpl2* ^*+/-*^ mice. *Ifnar1* ^*-/-*^ mice were kindly provided by Dr. Biao He (University of Georgia). Mice deficient in both IFNAR1 and Tpl2 were generated by interbreeding single knockout animals. To generate chimeric mice, WT or *Tpl2* ^*-/-*^ recipient mice (both CD45.2^+^) were lethally irradiated with 1100 rad and reconstituted with donor B6.SJL-*Ptprc* ^*a*^ *Pepc* ^*b*^/BoyJ (WT CD45.1^+^ congenic) or *Tpl2* ^*-/-*^ bone marrow cells. Chimeric mice were maintained for 8 weeks. Animals were housed in sterile microisolator cages in the Central Animal Facility of the College of Veterinary Medicine. Embryonated specific pathogen free (SPF) chicken eggs were purchased from Sunrise Farms, New York. Influenza viruses A/HKX31 (H3N2), A/Puerto Rico/8/34 (PR8; H1N1) and A/WSN/1933 (H1N1) stocks were propagated in the allantoic cavity of 9- to 11-day-old embryonated SPF chicken eggs at 37°C for 72 hr, and viral titers were enumerated by plaque assays [63].

### *In vivo* infections

Age-matched, 6- to 8-week-old WT, *Tpl2* ^*-/-*^, *Ifnar1* ^*-/-*^, *Ifnar1* ^*-/-*^ *Tpl2* ^*-/-*^ or chimeric mice were anesthetized with 250 mg/kg Avertin (2,2,2-tribromoethanol) followed by intranasal infection with influenza A/HK-X31 (H3N2) in 50 μl PBS. Control mice were mock-infected with a similar dilution of allantoic fluid. To determine lung viral titers, whole lungs from WT and *Tpl2* ^*-/-*^ mice infected with 10^4^ pfu of X31 virus were harvested on days 3, 5 and 7 pi. Lungs were placed in 1 ml PBS and dissociated with a bead mill homogenizer (Qiagen), and virus titers were enumerated by plaque assays. To assess susceptibility to influenza infection, WT and *Tpl2* ^*-/-*^ mice infected with 10^4^ pfu of X31 virus were observed over a period of 14 days. Body weights were recorded daily, and mice exhibiting severe signs of disease or more than 30% weight loss were euthanized. To measure IFN and cytokine secretion, mice infected with 10^6^ or 10^4^ pfu of X31 virus were euthanized 3 or 7 days pi, and bronchoalveolar lavage fluid (BALF) or BAL cells were obtained by washing the lungs twice with 1 mL PBS. Cells were recovered by centrifugation of the lavage fluid for 10 min at 250xg. BALF from the first wash was used for quantitation of cytokine secretion. Cellular recruitment was assessed by quantifying total leukocyte recovery from both washes.

### Measurement of antigen-specific CD8^+^ T cell responses

Mice infected with 10^4^ pfu of X31 virus were euthanized on day 10 pi, and cells were obtained by washing the lungs twice with 1 mL PBS. BAL cells were stained with anti-CD4, anti-CD8 (eBiosciences), and H2D^b^NP_366–374_ tetramer (NIH Tetramer Core Facility, Emory University, Atlanta, GA) for 30 min at 4°C and fixed in 1% formaldehyde. Cells were acquired on a BD LSRII flow cytometer and analyzed using FlowJo software (Tree Star, Inc.). For IFNγ measurement, BAL cells were stimulated with a cocktail of influenza immunodominant peptides (NP_366–374_, PA_224–233_, PB1_703–711_) (1 μg/mL) for 24 hr at 37°C, and IFNγ levels in culture supernatant was measured by ELISA (eBiosciences).

### Cell culture

Bone marrow derived macrophages (BMDMs), pDCs and mouse embryonic fibroblasts (MEFs) were generated from age- and sex-matched mice as described previously [17, 64]. CD11c^+^CD11b^-^B220^+^ pDCs were sorted using a Beckman Coulter MoFlo XDP cell sorter. In some experiments, cells were used on day 10 without sorting (referred as Flt3 ligand-derived DCs). Triggering of RIG-I was accomplished by directly delivering 5’-triphosphate RNA (5’ppp-RNA; 0.5 μg/mL) or a control RNA to the cytosol of BMDMs or MEFs using LyoVec transfection reagent (InvivoGen). 20 μL 5’ppp RNA or control RNA (100 μg/mL) was incubated with 200 μL LyoVec (62.5 μg/mL) at room temperature for 15 min to form complexes. Twenty-five microliters of the complexes were used to stimulate 2.5x10^5^ BMDMs or 0.5x10^5^ MEFs per well for 24 hr. BMDMs at 1x10^6^/mL were also treated with R848 (InvivoGen) (1 μg/mL) for 24 hr. To investigate IFN signaling, BMDMs at 1x10^6^/mL were treated with rmIFNα (2000 IU/mL; R&D Systems), or rhIFNβ (10 ng/mL; Peprotech) for 1–4 hr.

Plasmacytoid DCs at a concentration of 0.5-1x10^6^/mL were left untreated or stimulated with R848 (1 μg/mL), CpG ODN2395 (10 μg/mL) (InvivoGen), 50 ng/mL rhIFNβ (PeproTech) or infected with WSN virus at a MOI of 0.2 for 24 hr. In some experiments, cells were pretreated with LY294002 hydrochloride (20 μM), rapamycin (30 nM) or U0126 (20 μM) (Sigma) for 30 min before stimulating with CpG.

### Cytokine measurements

Cytokine levels were measured by ELISA (IFNα, IFNλ and IFNγ, eBioscience; IFNβ, PBL Interferon Source) or bead-based detection assays (Mouse IFNα Flowcytomix simplex, eBioscience; Mouse inflammation cytokine bead array, BD Biosciences).

### Analysis of mRNA expression

Cells stimulated with R848 or IFNs were lysed using TRK lysis buffer (Omega Bio-Tek). For *in vivo* infections, RNA lysates were prepared from tissue after homogenizing whole lungs. RNA was extracted using a Total RNA Kit (Omega Bio-Tek). Real-time PCR was performed after synthesizing cDNA using a High capacity cDNA Reverse Transcription kit (Applied Biosystems). The expression of *Irf7* (Mm00516791_g1), *Il28b (ifnl3)* (Mm00663660_g1), *Ifitm3* (Mm00847057_s1), *Isg15* (Mm01705338_s1), *Oasl2* (Mm00496187-m1), *Il12b* (Mm00434174_m1), *Il6* (Mm00446190_m1), *Tnfa* (Mm00443258_m1), *Ifna (Mm03030145-gH)*, *Ccl5 (Mm01302427-m1)* and *Actinb* (4352341E-1112017) were determined by RT-PCR (Applied Biosystems). RT-PCR reactions were performed in microAmp Fast plates (Applied Biosystems) using SensiFAST Probe Hi-ROX kit (Bioline) and a StepOnePlus RT-PCR machine (Applied Biosystems). Relative gene expression levels were calculated by normalizing the Ct levels of the target gene to both endogenous actin levels and an unstimulated WT control using the ΔΔCt method.

### Protein analysis

Cell lysates were separated on 4–12% gradient gels (Invitrogen) and were transferred to PVDF membranes using the iBlot Gel Transfer system (Invitrogen). Membranes were probed with various antibodies followed by horseradish peroxidase (HRP)-labeled secondary antibodies. Protein bands were visualized by enhanced chemiluminescent reagent (Lumigen) and Amersham Hyperfilm ECL (GE Healthcare). The following antibodies were used for immunoblotting: Tpl2 (Cot M-20), ERK1, ERK2 and β-actin (Santa Cruz Biotechnology), p-ERK1/2 (Thr202/Tyr204), p-STAT1 (Tyr701), p-STAT1 (Ser727) and STAT1 (Cell Signaling Technology).

### Intracellular staining

Cells harvested after overnight stimulation were fixed, permeabilized with triton buffer (PBS+0.5%triton+0.1%BSA) and stained for p-Akt (Ser473) according to manufacturers’ protocol (Cell Signaling Technology). Samples were acquired on a BD LSRII flow cytometer and analyzed using FlowJo software (Tree Star, Inc.).

### Statistical analysis

Data represent means ± SEM, except where indicated. P-values were determined by Students *t*-test, and significance was assigned for p-values <0.05. Kaplan-Meier analysis using PRISM software was performed to estimate percentage survival of WT and *Tpl2* ^*-/-*^ groups infected with influenza virus, and *p* value was determined using a Mantel-Cox test.

## Supporting Information

S1 Fig Virus replication is increased in *Tpl2* ^*-/-*^ littermate control mice. WT and *Tpl2* ^*-/-*^ littermate mice were infected intranasally with 10^4^ pfu of X31 virus, and lung viral titers were enumerated by plaque assay on D7 pi; n = 4 WT and 3 *Tpl2* ^*-/-*^. ** indicates *p*<0.01.
(TIF)

S2 Fig Lung viral titers are similar between WT and *Tpl2* ^*-/-*^ mice at day 1 post infection. WT and *Tpl2* ^*-/-*^ mice were intranasally infected with 10^6^ pfu of X31 virus, and lung viral titers were enumerated by plaque assay on D1 pi; n = 3 WT and 3 *Tpl2* ^*-/-*^ mice.
(TIF)

S3 Fig Inflammatory responses are increased in the lungs of *Tpl2* ^*-/-*^ mice early after infection. WT and *Tpl2* ^*-/-*^ mice were infected with 10^6^ pfu of X31 virus, and the number of cells recovered (A) and cytokine levels in BALF (B) were assessed on D3 pi; n = 6 uninfected and 12 (WT) and 10 (*Tpl2* ^*-/-*^) infected. ** indicates *p*<0.01.
(TIF)

S4 Fig Recruitment of pDCs to lungs during influenza virus infection occurs independently of Tpl2. Animals infected with 10^6^ pfu of X31 virus were anesthetized with a lethal dose of avertin, and lungs were perfused with 25 mL PBS/heparin sodium solution. Harvested lungs were minced and incubated in 1.25 mM EDTA for 30 min at 37°C. The tissue was further incubated in collagenase diluted in RPMI (6 mg/mL) at 37°C for 30 min. Supernatants from digestions were passed through a 70 μm cell strainer. Cells were enriched by Percoll (GE Healthcare) gradient purification using a 47/67% gradient. Cells at the interface were collected and stained with antibody cocktail containing anti-CD16/32, CD11c-PE, PDCA1-APC, CD8-eFlour, B220-FITC and TCRβ-PerCP-Cy5.5 for 15 min at 4°C and fixed in PBS containing 1% formaldehyde. Samples were run on a BD LSRII flow cytometer and analyzed using FlowJo software (Tree Star, Inc.). (A) Representative flow plots. (B) Proportions and (C) absolute numbers of B220^+^PDCA1^+^ cells; n = 1 uninfected and 3 infected WT and *Tpl2* ^*-/-*^ mice. (D) WT and *Tpl2* ^*-/-*^ mice were infected with 10^6^ pfu of X31 virus, and the expression of *Pdca1* in lung tissue D1 or D3 pi was measured by RT-PCR with normalization to actin mRNA and WT uninfected sample (D1, n = 7; D3, n = 5).
(TIF)

S5 Fig Tpl2 is required for IFNλ induction in pDCs infected with X31 influenza virus. Flt3 ligand-derived DCs from WT and *Tpl2* ^*-/-*^ mice were infected with X31 or WSN virus at an MOI of 10 or stimulated with CpG for 24 hr. IFNλ3 (*Il28b*) expression was measured by RT-PCR with normalization to actin mRNA and WT uninfected sample. Data are representative of three independent experiments.
(TIF)

S6 Fig ERK phosphorylation is impaired in Tpl2-deficient BMDMs stimulated with model viral ligands. BMDMs from WT and *Tpl2* ^*-/-*^ mice were left untreated or stimulated with TLR or RLR ligands for 2 hr, and ERK phosphorylation was assessed by immunoblotting. Data are representative of three independent experiments.
(TIF)

S7 Fig Tpl2-dependent induction of IFNλ occurs early after stimulation like other NFκB-regulated proinflammatory cytokines. Plasmacytoid DCs from WT and *Tpl2* ^*-/-*^ mice were left untreated or stimulated with R848 for 2 hr, and expression of IFNλ3 (*Il28b*), *Ifna*, *Irf7*, *Il12p40*, *Tnfa*, and *Ccl5* were measured by RT-PCR relative to an actin control and WT untreated sample. Data are representative of two independent experiments.
(TIF)

S8 Fig ERK and mTOR/Akt regulation of IFNλ induction is more pronounced in WT compared to *Tpl2* ^*-/-*^ pDCs. Flt3 ligand-derived DCs from WT and *Tpl2* ^*-/-*^ mice were pretreated with inhibitors for 30 min prior to stimulation with CpG for 24 hr. IFNλ3 (*Il28b*) expression was measured by RT-PCR relative to an actin control and WT untreated sample. Data are from three independent experiments. ** indicates p<0.01 (Bonferroni multiple comparison test).
(TIF)

S9 Fig Tpl2 is induced in response to Type I IFNs and influenza virus infection. (A) BMDMs from WT mice were stimulated with IFNβ, and Tpl2 (*Map3k8)* gene expression was measured by RT-PCR with normalization to endogenous actin mRNA and the WT untreated control. Data are pooled from 3 independent experiments. (B) pDCs from WT mice were infected with WSN virus at an MOI of 0.2 for 4 or 24 hr, and *Map3k8* expression was measured by RT-PCR. (C) WT mice were infected with 10^6^ pfu of X31 virus, and the expression of *Map3k8* in lung tissue D1 pi was measured by RT-PCR with normalization to actin mRNA and WT uninfected sample; n = 2 uninfected and 7 infected mice.
(TIF)

S10 Fig Tpl2 regulates the induction of ISGs in BMDMs in response to IFNβ. BMDMs were treated with IFNβ, and expression of *Ifitm3*, *Isg15* and *Oasl2* were measured by RT-PCR with normalization to actin mRNA and WT untreated control. Data are pooled from 3 independent experiments.
(TIF)

S11 Fig Increased susceptibility to infection and decreased antigen-specific CD8^+^ T cell responses in *Tpl2* ^*-/-*^ mice infected with A/Puerto Rico/8/34 (PR8; H1N1). (A) WT and *Tpl2* ^*-/-*^ mice were infected with 30 pfu of PR8 virus, and body weights were recorded daily for 10 days. At 10 days pi the experiment was halted due to severe clinical signs in Tpl2-deficient mice, and serum cytokine levels and antigen-specific recall responses were evaluated. (B) Levels of inflammatory cytokines in serum samples collected on D10 pi were measured by cytokine bead array. (C) BAL cells were collected by lung lavage. Cells were enumerated and stimulated with NP specific peptide. Cells were fixed, permeabilized, and stained using anti-mouse CD8 and IFNγ monoclonal antibodies. Samples were acquired on a BD LSRII flow cytometer and analyzed using FlowJo software (Tree Star, Inc.). The proportions of IFNγ^+^ cells in WT and *Tpl2* ^*-/-*^ BAL samples are shown. * indicates *p*<0.05.
(TIF)
