# Supplementary figures and images for: Tumor Progression Locus 2 Promotes Induction of IFNλ, Interferon Stimulated Genes and Antigen-Specific CD8+ T Cell Responses and Protects against Influenza Virus
Source: PLoS Pathog. 2015 Aug 4;11(8):e1005038. doi: 10.1371/journal.ppat.1005038 (PMC4524623; doi:10.1371/journal.ppat.1005038)

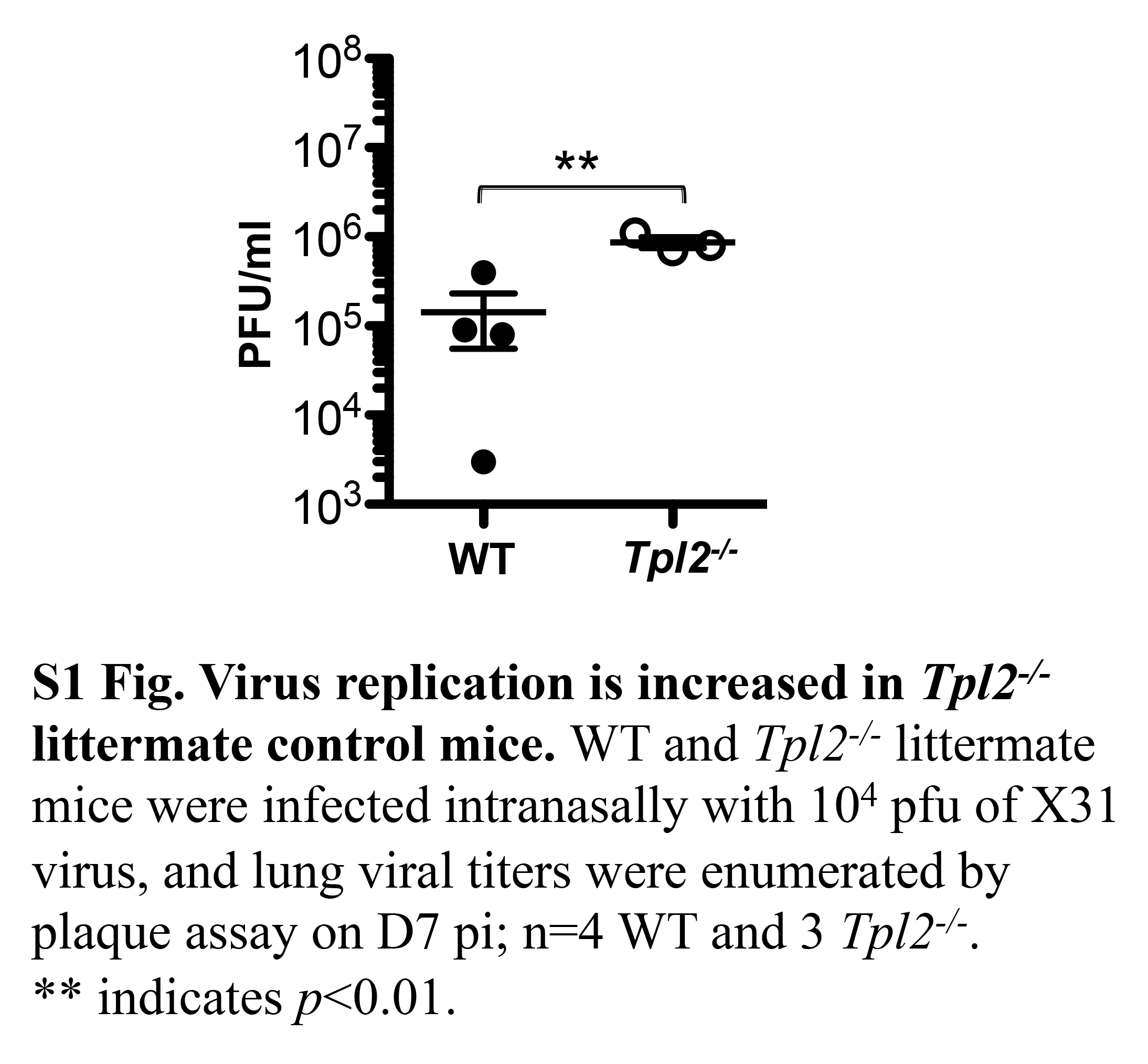

Supplement: S1 Fig — WT and Tpl2 -/- littermate mice were infected intranasally with 104 pfu of X31 virus, and lung viral titers were enumerated by plaque assay on D7 pi; n = 4 WT and 3 Tpl2 -/-. ** indicates p<0.01. (TIF) [file ppat.1005038.s001.tif]

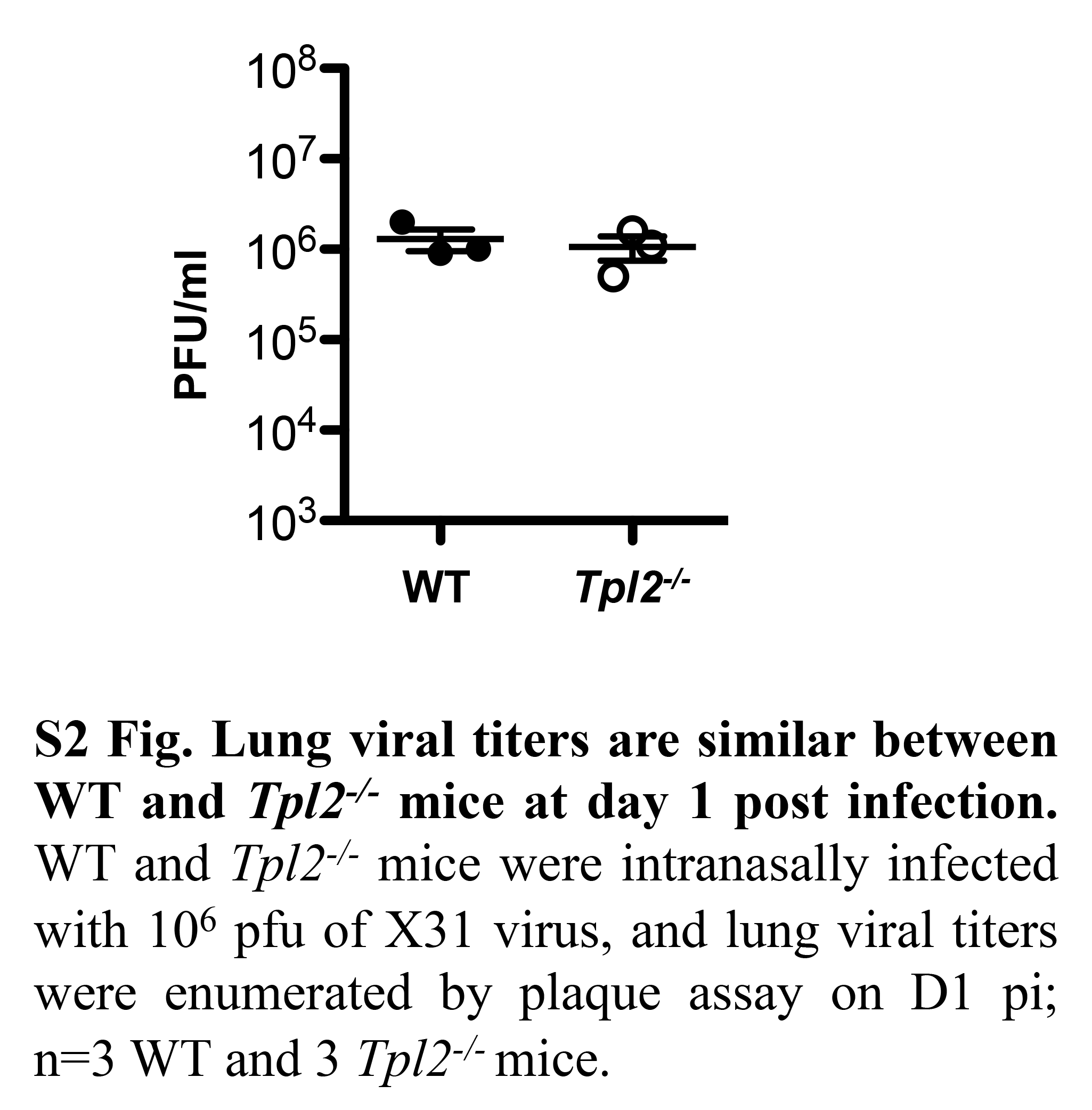

Supplement: S2 Fig — WT and Tpl2 -/- mice were intranasally infected with 106 pfu of X31 virus, and lung viral titers were enumerated by plaque assay on D1 pi; n = 3 WT and 3 Tpl2 -/- mice. (TIF) [file ppat.1005038.s002.tif]

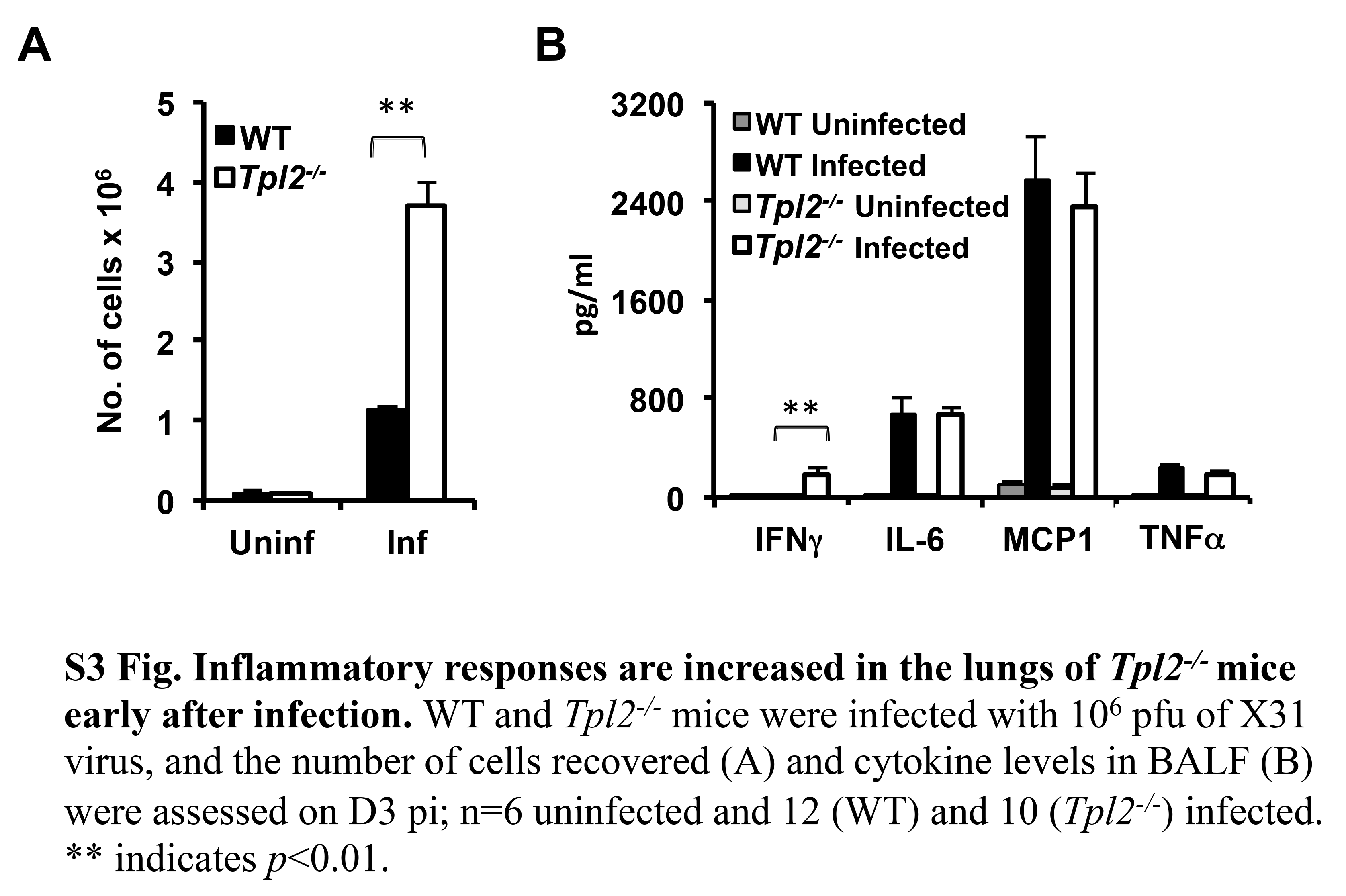

Supplement: S3 Fig — WT and Tpl2 -/- mice were infected with 106 pfu of X31 virus, and the number of cells recovered (A) and cytokine levels in BALF (B) were assessed on D3 pi; n = 6 uninfected and 12 (WT) and 10 (Tpl2 -/-) infected. ** indicates p<0.01. (TIF) [file ppat.1005038.s003.tif]

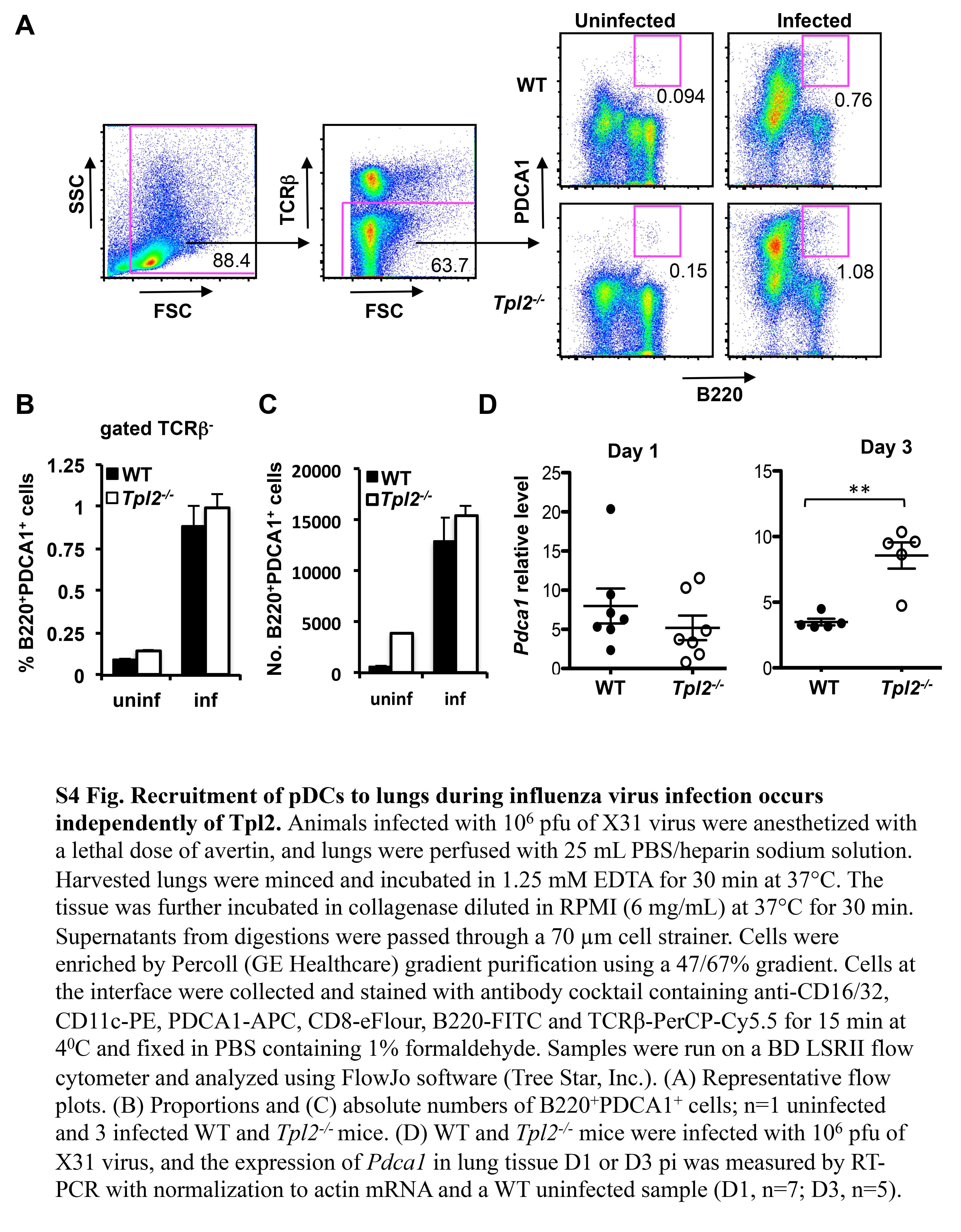

Supplement: S4 Fig — Animals infected with 106 pfu of X31 virus were anesthetized with a lethal dose of avertin, and lungs were perfused with 25 mL PBS/heparin sodium solution. Harvested lungs were minced and incubated in 1.25 mM EDTA for 30 min at 37°C. The tissue was further incubated in collagenase diluted in RPMI (6 mg/mL) at 37°C for 30 min. Supernatants from digestions were passed through a 70 μm cell strainer. Cells were enriched by Percoll (GE Healthcare) gradient purification using a 47/67% gradient. Cells at the interface were collected and stained with antibody cocktail containing anti-CD16/32, CD11c-PE, PDCA1-APC, CD8-eFlour, B220-FITC and TCRβ-PerCP-Cy5.5 for 15 min at 4°C and fixed in PBS containing 1% formaldehyde. Samples were run on a BD LSRII flow cytometer and analyzed using FlowJo software (Tree Star, Inc.). (A) Representative flow plots. (B) Proportions and (C) absolute numbers of B220+PDCA1+ cells; n = 1 uninfected and 3 infected WT and Tpl2 -/- mice. (D) WT and Tpl2 -/- mice were infected with 106 pfu of X31 virus, and the expression of Pdca1 in lung tissue D1 or D3 pi was measured by RT-PCR with normalization to actin mRNA and WT uninfected sample (D1, n = 7; D3, n = 5). (TIF) [file ppat.1005038.s004.tif]

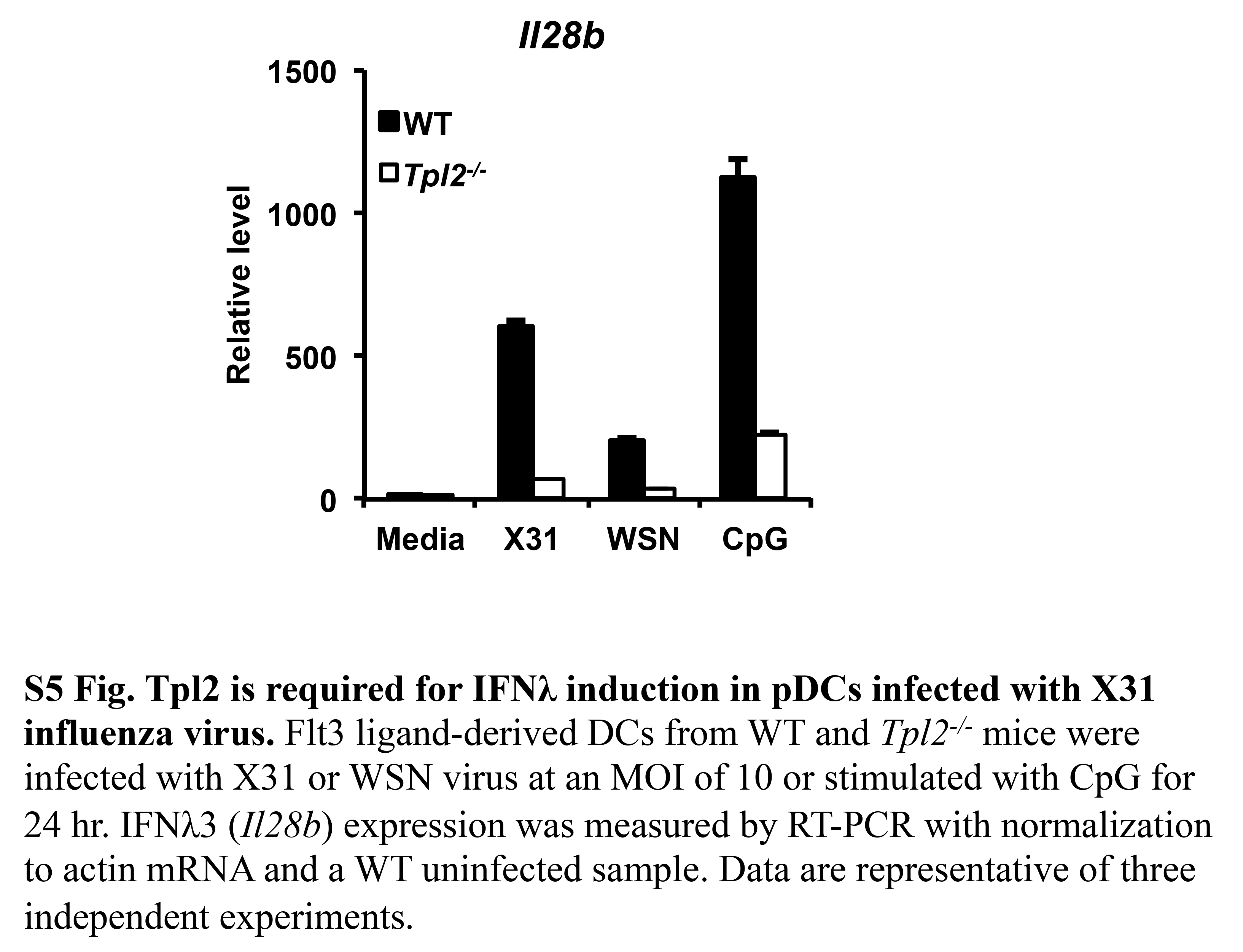

Supplement: S5 Fig — Flt3 ligand-derived DCs from WT and Tpl2 -/- mice were infected with X31 or WSN virus at an MOI of 10 or stimulated with CpG for 24 hr. IFNλ3 (Il28b) expression was measured by RT-PCR with normalization to actin mRNA and WT uninfected sample. Data are representative of three independent experiments. (TIF) [file ppat.1005038.s005.tif]

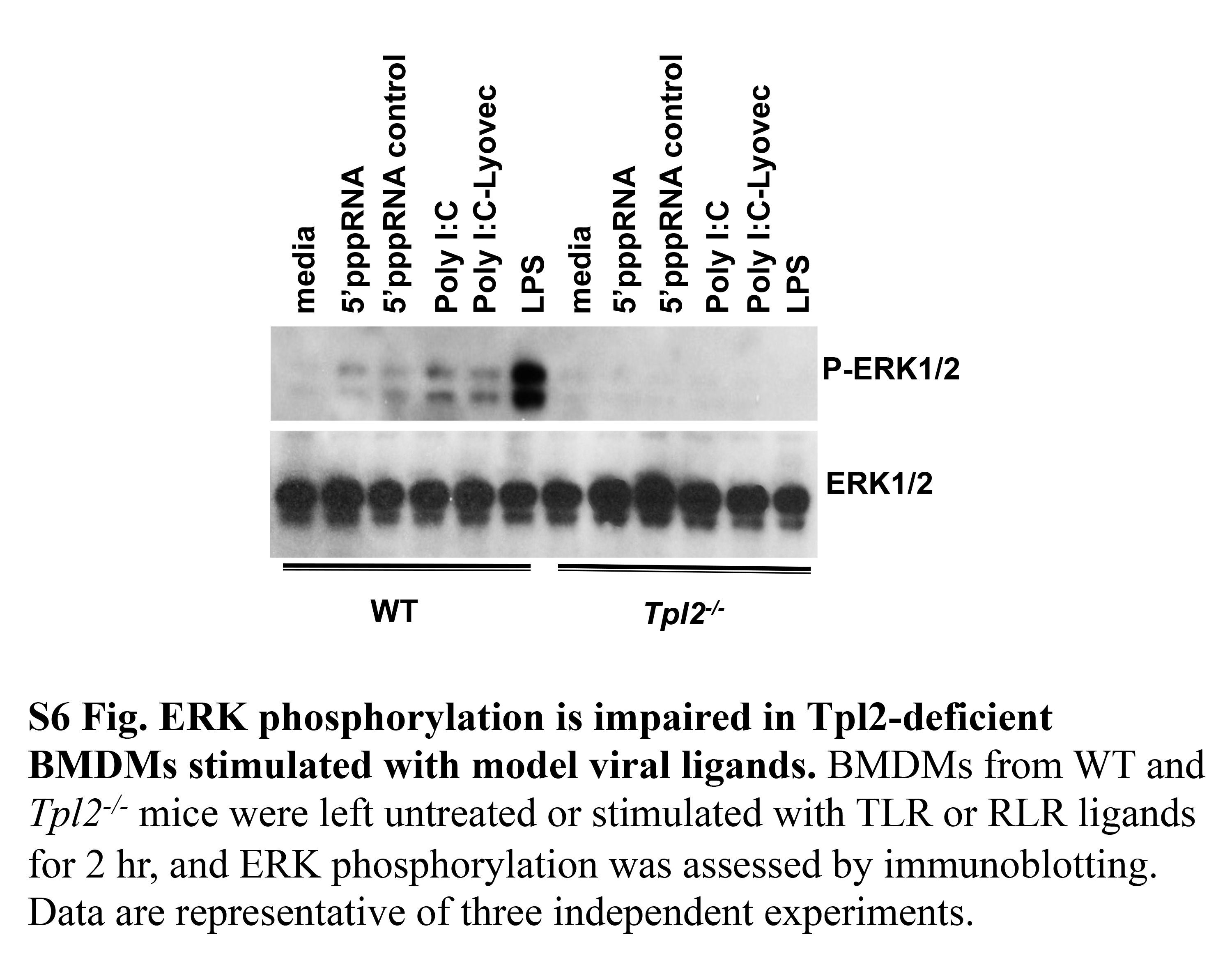

Supplement: S6 Fig — BMDMs from WT and Tpl2 -/- mice were left untreated or stimulated with TLR or RLR ligands for 2 hr, and ERK phosphorylation was assessed by immunoblotting. Data are representative of three independent experiments. (TIF) [file ppat.1005038.s006.tif]

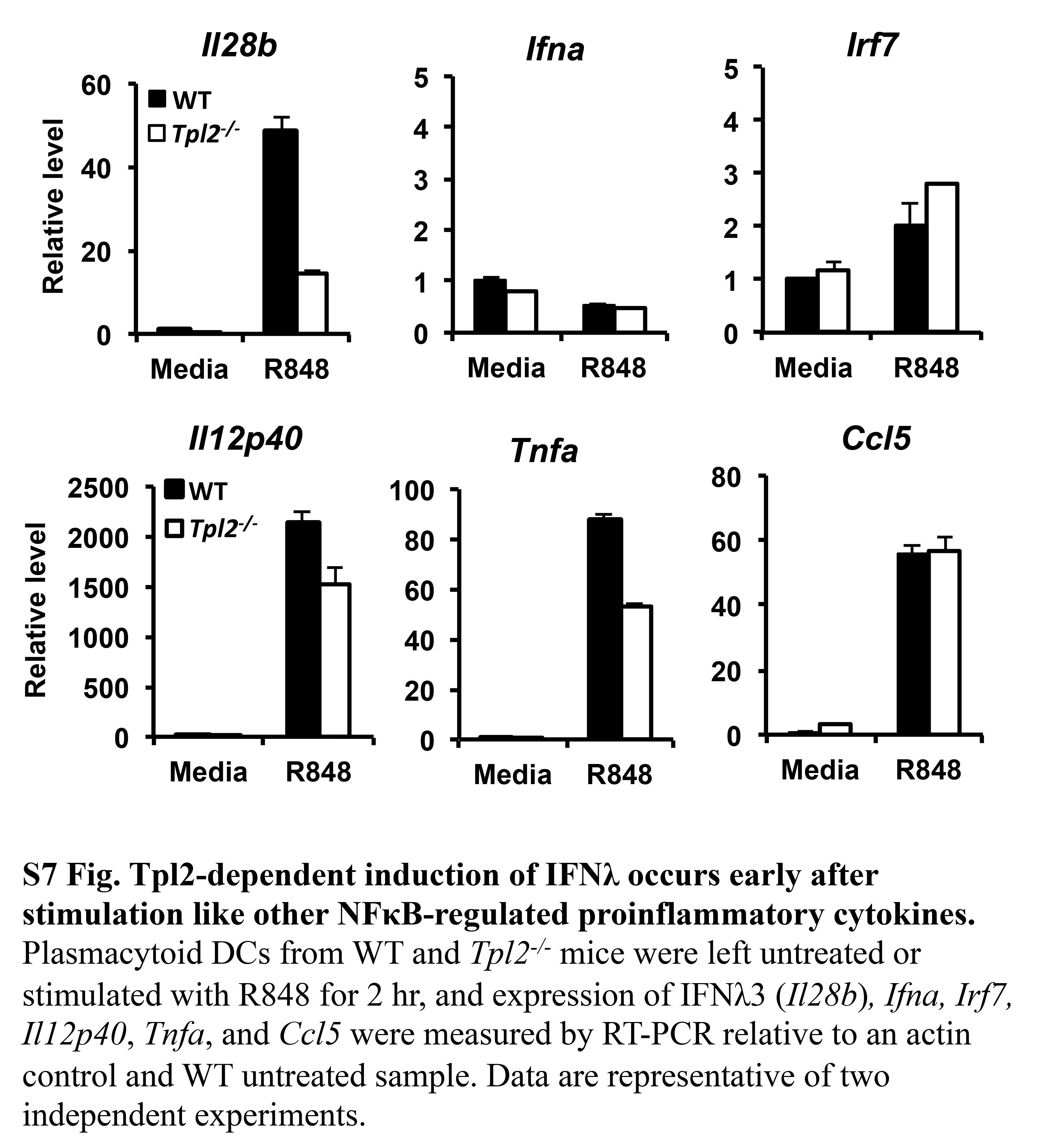

Supplement: S7 Fig — Plasmacytoid DCs from WT and Tpl2 -/- mice were left untreated or stimulated with R848 for 2 hr, and expression of IFNλ3 (Il28b), Ifna, Irf7, Il12p40, Tnfa, and Ccl5 were measured by RT-PCR relative to an actin control and WT untreated sample. Data are representative of two independent experiments. (TIF) [file ppat.1005038.s007.tif]

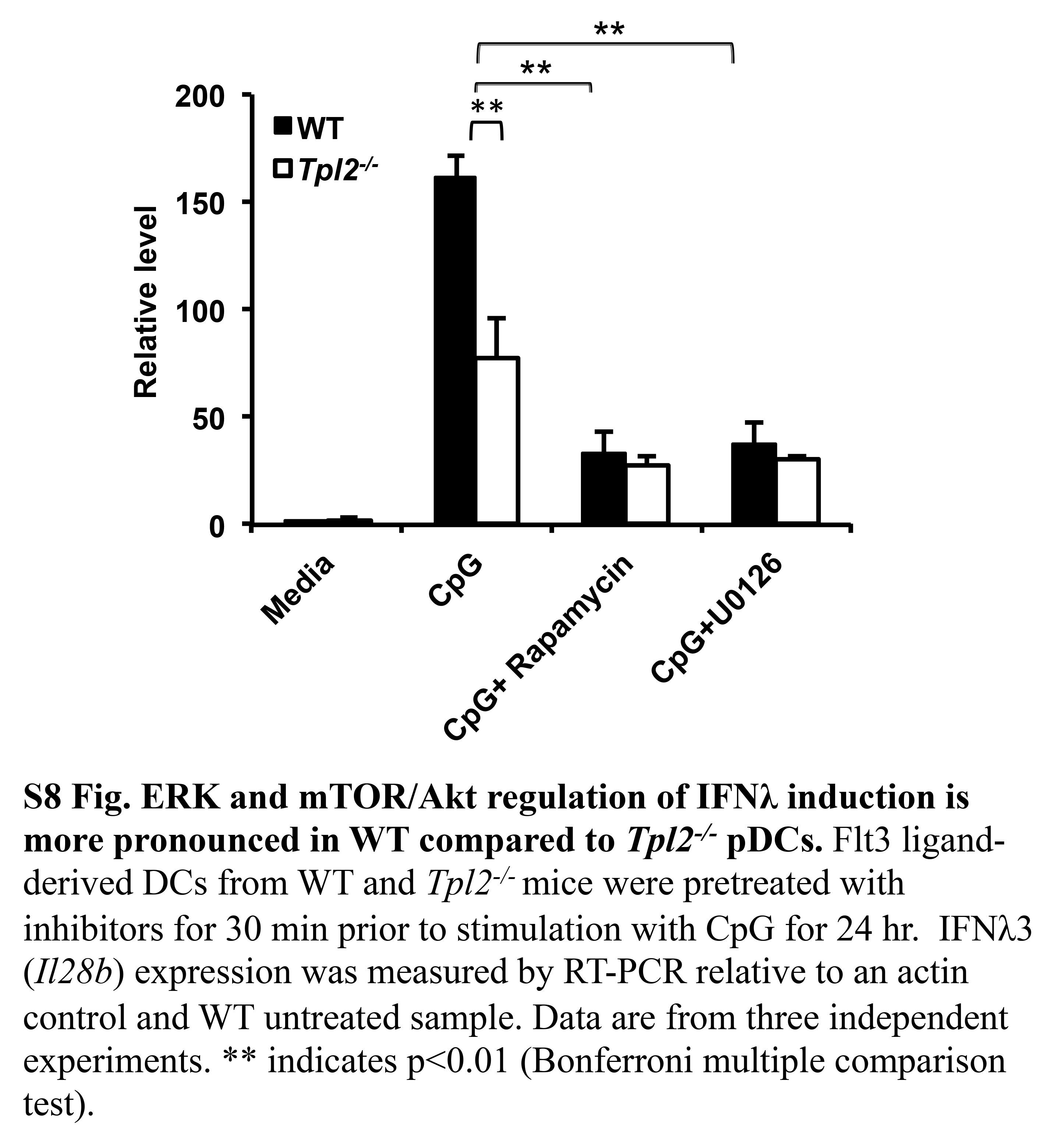

Supplement: S8 Fig — Flt3 ligand-derived DCs from WT and Tpl2 -/- mice were pretreated with inhibitors for 30 min prior to stimulation with CpG for 24 hr. IFNλ3 (Il28b) expression was measured by RT-PCR relative to an actin control and WT untreated sample. Data are from three independent experiments. ** indicates p<0.01 (Bonferroni multiple comparison test). (TIF) [file ppat.1005038.s008.tif]

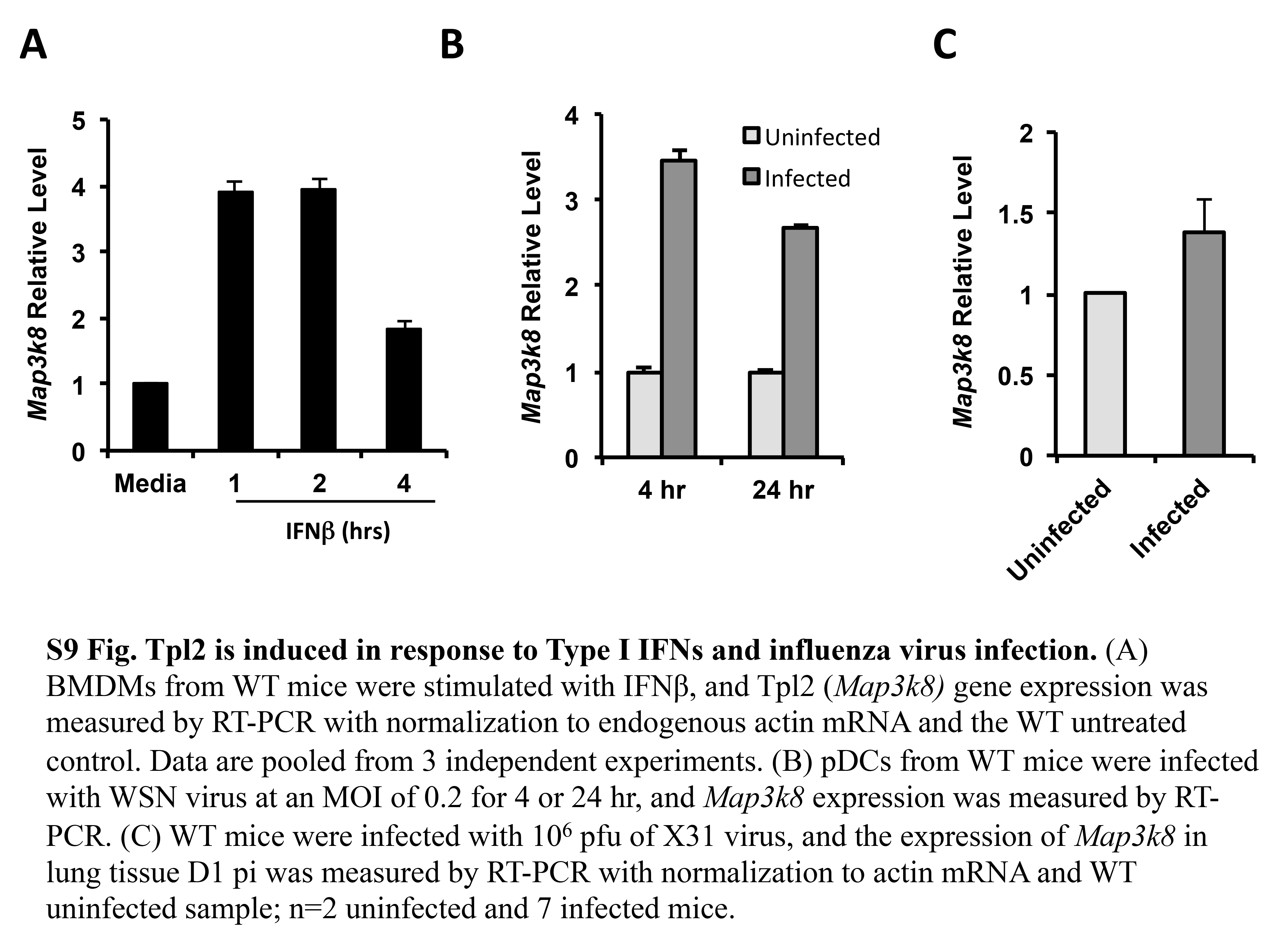

Supplement: S9 Fig — (A) BMDMs from WT mice were stimulated with IFNβ, and Tpl2 (Map3k8) gene expression was measured by RT-PCR with normalization to endogenous actin mRNA and the WT untreated control. Data are pooled from 3 independent experiments. (B) pDCs from WT mice were infected with WSN virus at an MOI of 0.2 for 4 or 24 hr, and Map3k8 expression was measured by RT-PCR. (C) WT mice were infected with 106 pfu of X31 virus, and the expression of Map3k8 in lung tissue D1 pi was measured by RT-PCR with normalization to actin mRNA and WT uninfected sample; n = 2 uninfected and 7 infected mice. (TIF) [file ppat.1005038.s009.tif]

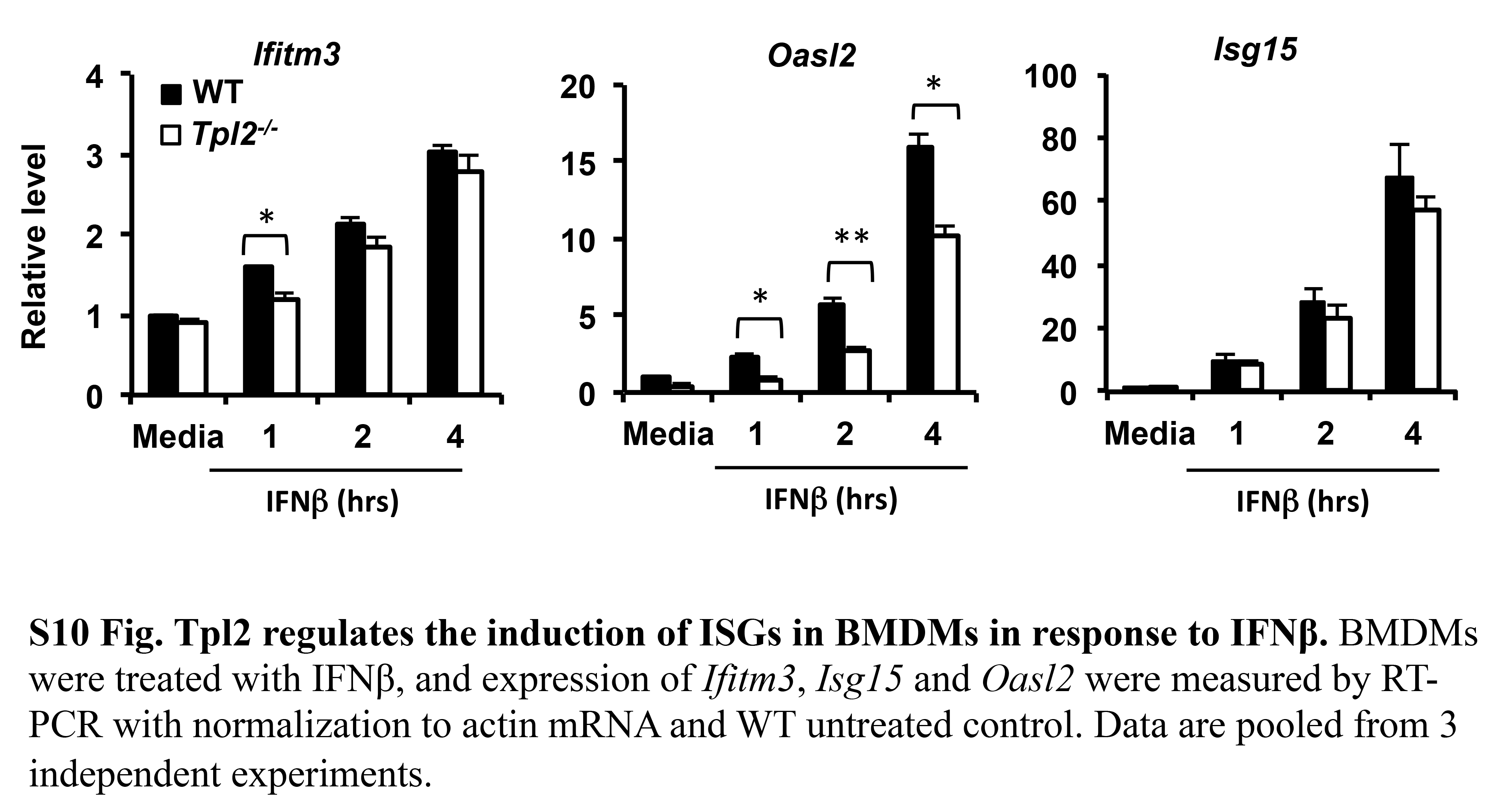

Supplement: S10 Fig — BMDMs were treated with IFNβ, and expression of Ifitm3, Isg15 and Oasl2 were measured by RT-PCR with normalization to actin mRNA and WT untreated control. Data are pooled from 3 independent experiments. (TIF) [file ppat.1005038.s010.tif]

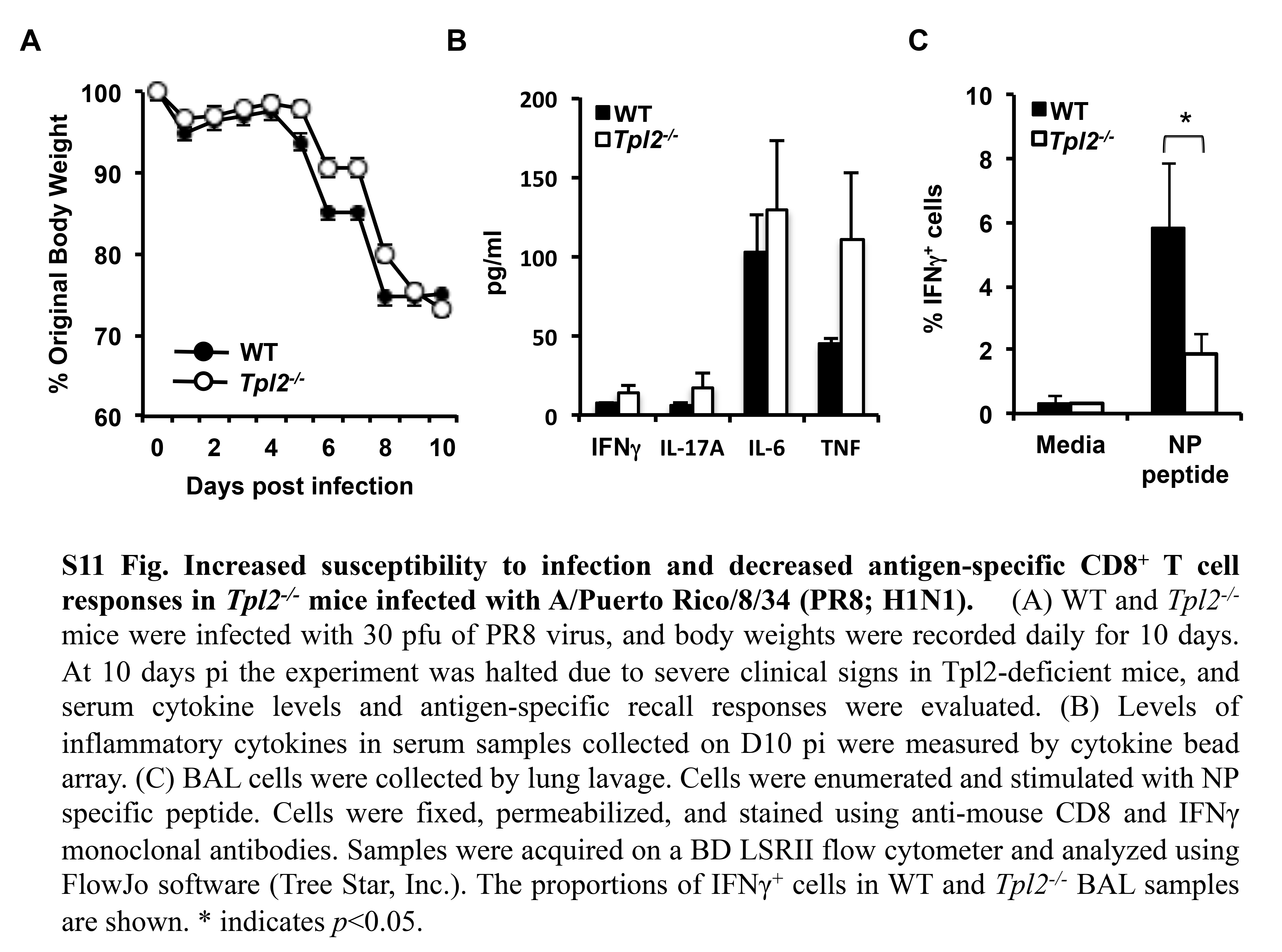

Supplement: S11 Fig — (A) WT and Tpl2 -/- mice were infected with 30 pfu of PR8 virus, and body weights were recorded daily for 10 days. At 10 days pi the experiment was halted due to severe clinical signs in Tpl2-deficient mice, and serum cytokine levels and antigen-specific recall responses were evaluated. (B) Levels of inflammatory cytokines in serum samples collected on D10 pi were measured by cytokine bead array. (C) BAL cells were collected by lung lavage. Cells were enumerated and stimulated with NP specific peptide. Cells were fixed, permeabilized, and stained using anti-mouse CD8 and IFNγ monoclonal antibodies. Samples were acquired on a BD LSRII flow cytometer and analyzed using FlowJo software (Tree Star, Inc.). The proportions of IFNγ+ cells in WT and Tpl2 -/- BAL samples are shown. * indicates p<0.05. (TIF) [file ppat.1005038.s011.tif]
